# A brain-permeable inhibitor of the neurodegenerative disease target kynurenine 3-monooxygenase prevents accumulation of neurotoxic metabolites

**DOI:** 10.1038/s42003-019-0520-5

**Published:** 2019-07-24

**Authors:** Shaowei Zhang, Michiyo Sakuma, Girdhar S. Deora, Colin W. Levy, Alex Klausing, Carlo Breda, Kevin D. Read, Chris D. Edlin, Benjamin P. Ross, Marina Wright Muelas, Philip J. Day, Stephen O’Hagan, Douglas B. Kell, Robert Schwarcz, David Leys, Derren J. Heyes, Flaviano Giorgini, Nigel S. Scrutton

**Affiliations:** 1 Manchester Institute of Biotechnology and School of Chemistry, The University of Manchester, Manchester, M1 7DN UK; 2 School of Pharmacy, The University of Queensland, Brisbane, Queensland 4072 Australia; 3 Maryland Psychiatric Research Center, University of Maryland School of Medicine, Baltimore, MD 21228 USA; 4 Department of Genetics and Genome Biology, University of Leicester, Leicester, LE1 7RH UK; 5 Drug Discovery Unit, School of Life Sciences, University of Dundee, Dundee, Scotland DD1 5EH UK; 6 Teva Pharmaceuticals, Waterford, X91 WK68 Ireland; 7 Manchester Institute of Biotechnology and Faculty of Biology, Medicine and Health, The University of Manchester, Manchester, M13 9PL UK

**Keywords:** Drug discovery, X-ray crystallography, Chemical biology, Neurodegeneration

## Abstract

Dysregulation of the kynurenine pathway (KP) leads to imbalances in neuroactive metabolites associated with the pathogenesis of several neurodegenerative disorders, including Huntington’s disease (HD). Inhibition of the enzyme kynurenine 3-monooxygenase (KMO) in the KP normalises these metabolic imbalances and ameliorates neurodegeneration and related phenotypes in several neurodegenerative disease models. KMO is thus a promising candidate drug target for these disorders, but known inhibitors are not brain permeable. Here, 19 new KMO inhibitors have been identified. One of these (**1**) is neuroprotective in a *Drosophila* HD model but is minimally brain penetrant in mice. The prodrug variant (**1b**) crosses the blood–brain barrier, releases **1** in the brain, thereby lowering levels of 3-hydroxykynurenine, a toxic KP metabolite linked to neurodegeneration. Prodrug **1b** will advance development of targeted therapies against multiple neurodegenerative and neuroinflammatory diseases in which KP likely plays a role, including HD, Alzheimer’s disease, and Parkinson’s disease.

## Abstract (web summary)

Kynurenine 3-monooxygenase (KMO) regulates levels of neuroactive kynurenine pathway metabolites associated with neurodegeneration. Its inhibition ameliorates disease phenotypes in animal models but current inhibitors are not brain permeable. Through structure-based virtual screening and compound synthesis, Zhang et al. now develop KMO inhibitors that can enter the rodent brain to modulate metabolism in this pathway.

## Introduction

**Fig. 1:**
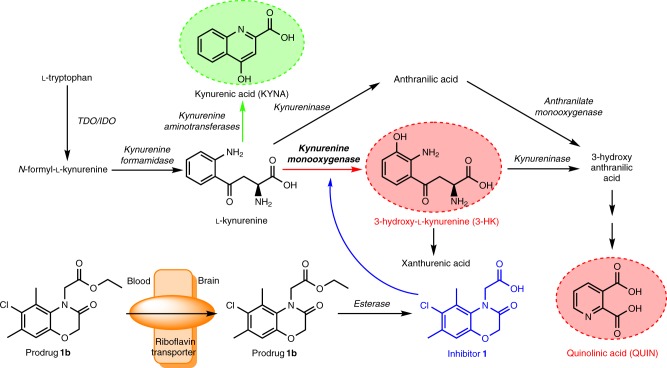
The kynurenine pathway of tryptophan metabolism. Enzymes are indicated in italics. The neurotoxic metabolites 3-HK and QUIN are highlighted in red and the neuroprotective metabolite KYNA in green. The prodrug **1b** is transported across the blood–brain barrier via riboflavin transporters, where it is converted, and thereby releases **1**. TDO, tryptophan 2,3-dioxygenase. IDO, indoleamine-2,3-dioxygenase

The kynurenine pathway (KP) (Fig. 1) degrades >95% of l-tryptophan in mammals and produces neuroactive metabolites that modulate neurodegeneration and associated phenotypes in models of several disorders^1–7^. Indeed, studies indicate that KP metabolite imbalances leading to elevated levels of the free-radical generator 3-hydroxykynurenine (3-HK) and the excitotoxin quinolinic acid (QUIN) relative to the neuroprotective metabolite kynurenic acid (KYNA) (Fig. 1) contribute to pathogenesis^8^. Upregulation of the central neurotoxic branch of the KP is linked to expression of proinflammatory cytokines during inflammation, which increases expression of KMO and indoleamine-2, 3-dioxygenase 1^9^. Downregulation of KMO activity shifts the flux between the two branches of the KP toward increased KYNA formation, thus generating a neuroprotective environment (Fig. 1)^10^. In fact, KMO inhibition ameliorates disease phenotypes in fly and mouse models of several neurodegenerative disorders^3–5,7,11^. KMO inhibitors are therefore increasingly considered for strategies to prevent or arrest neurodegeneration^9^ and as tools to experimentally modulate KP metabolism^4,11–17^. However, it should be noted, that complete elimination of metabolites downstream of KMO is not desirable, as loss-of-function mutations in downstream enzymes cause congenital malformations in humans and mice^18^. Thus, when targeting the KP, one needs to consider normalisation of metabolite imbalances arising from disease, and not depletion of key metabolites. Notably, existing KMO inhibitors do not appreciably enter the brain after systemic administration^11^. Rather, they act in the periphery, raising levels of l-KYN in the blood. l-KYN is then actively transported across the blood–brain barrier and preferentially converted to neuroprotective KYNA in the central nervous system (CNS)^4,11^.

Despite considerable efforts, the development of brain-permeable KMO inhibitors for experimental and possible clinical use has not been successful thus far. Such compounds would be highly desirable as KMO inhibition within the CNS, in contrast to selective enzyme inhibition in the periphery, results in a substantive reduction of 3-HK in the brain^17,19^. For drugs to pass effectively through cell membranes (including the blood–brain barrier) solute carrier (SLC)-type transporters are advantageous^20,21^. Thus, one strategy for identifying brain-penetrant compounds is to understand, which endogenous molecules drugs most resemble^22^. Here, we used structure-based virtual screening and compound synthesis alongside cheminformatics to identify brain permeable, drug-like KMO inhibitors, and to assess their transport mechanism. These insights led to the development of a prodrug (**1b**) that releases a neuroprotective KMO inhibitor within the CNS after peripheral application and which then directly modulates KP metabolite levels in the brain.

## Results

### Virtual screening identifies novel KMO inhibitors

A combination of ligand- and structural-based virtual screening approaches (see supporting information for details) was used to identify an initial library of >1000 compounds that may be suitable as potential KMO inhibitors. These compounds were screened for inhibition of *Pseudomonas fluorescens* KMO (*Pf*KMO) and *Homo sapiens* KMO (*Hs*KMO) using high-throughput assays by monitoring the time-dependent oxidation of NADPH at 340 nm^23^. This yielded a total of 19 new inhibitor compounds (IC_50_ ≤ 200 µM) (Table 1). The corresponding structures reveal that the majority retained a carboxylate moiety that forms interactions with KMO active-site residues Arg83 and Tyr97 (residues were labelled according to *Pf*KMO) which are essential for the binding of previously identified inhibitor molecules^23^. The new compounds inhibited both *Pf*KMO and *Hs*KMO with IC_50_ values ranging from 400 nM to 400 µM (Table 1 and Supplementary Fig. 1).

**Table 1:**
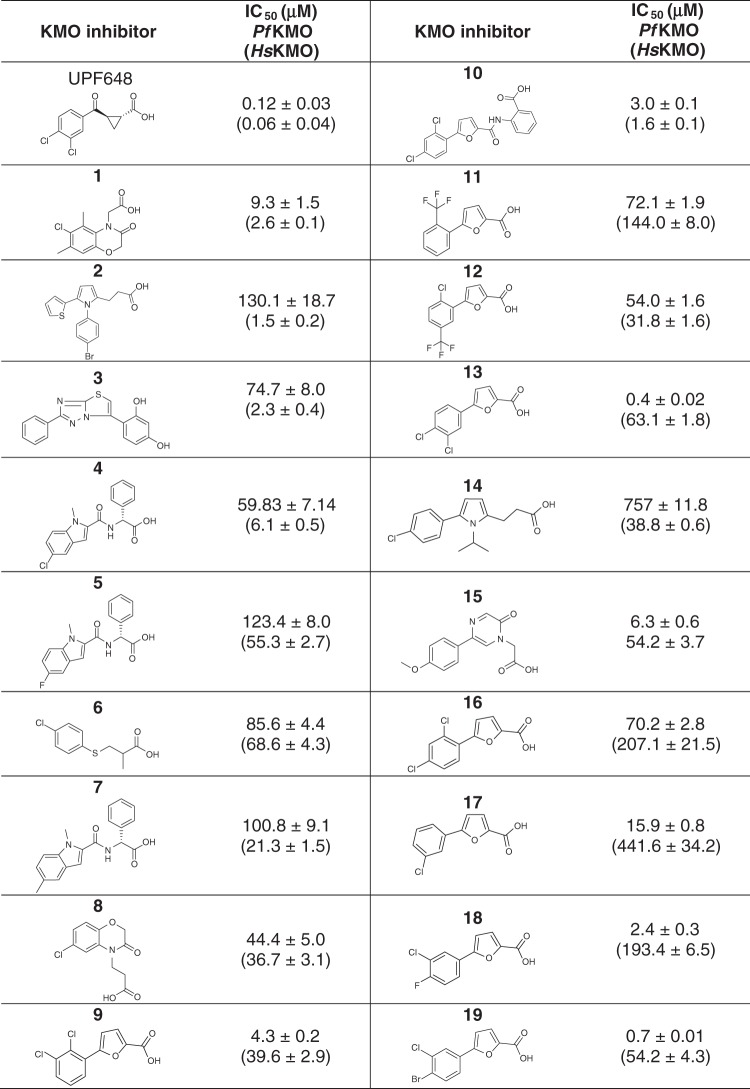
Structures and IC_50_ values of KMO inhibitor compounds

Compounds 9, 11–13 and 16–19 are structurally similar to the KMO inhibitor 6-(3,4-dichlorophenyl) pyrimidine-4-carboxylic acid^13^, in which the pyrimidine ring of the reported inhibitor is replaced by an isosteric furan ring in 9, 11–13 and 16–19. Compound 8 has been described previously as an inhibitor of KMO^32^

**Fig. 2:**
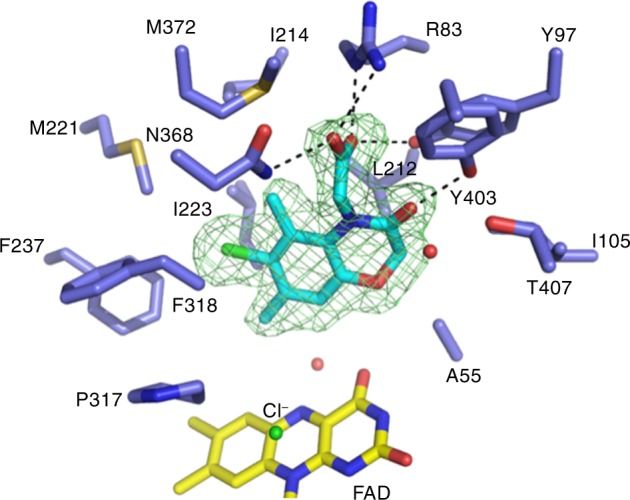
Structure of the *Pf*KMO active site in complex with **1**. Key active site residues are shown in atom coloured sticks (carbons of *Pf*KMO in blue, **1** in cyan and the FAD in yellow). Omit electron density map for bound inhibitor is shown as a green mesh contoured at three sigma. Hydrogen bonding interactions established between inhibitor and protein are shown as black dashed lines. All residues shown are conserved in human KMO

We next determined crystal structures of KMO in complex with the novel inhibitors or with l-KYN to inform the design of small-molecule inhibitors capable of penetrating the blood–brain barrier. We conjectured that the crystal structures would inform on which regions of the inhibitor could be modified as part of a prodrug strategy to enable transport across the blood–brain barrier. Co-crystallisation studies of *Pf*KMO with l-KYN or with the test compounds yielded crystal structures for the KMO-l-KYN complex (Supplementary Fig. 2; Protein Data Bank (PDB) code 6FOX) and four KMO inhibitor complexes (**1**, **4**, **9** and **13**; Fig. 2 and Supplementary Fig. 3; PDB codes 6FP1, 6FP0, 6FOY and 6FOZ). In each case, the inhibitor was bound in the l-KYN-binding pocket, with the chlorinated aromatic moiety occupying a similar position to the substrate l-KYN aniline group. Furthermore, the carboxylate group of the inhibitor was in close proximity to *Pf*KMO R83/Y97/N368. However, only compound **1** established a network of polar interactions that closely resembled the enzyme–substrate contacts. This both rationalises the respective affinity for each of these compounds and reflects the remarkable malleability of the KMO active site.

The structure of the KMO-l-KYN complex was determined at a resolution of 1.9Å (PDB code 6FOX). The l-KYN substrate is clearly bound in only one of the KMO monomers in the asymmetric unit, and resembles the previously reported KMO: l-KYN complex (PDB 5NAK; Supplementary Fig. 2). However, there are some differences in the protein:substrate interaction network, especially surrounding the substrate carboxylate group. Additional hydrogen bonding interactions are observed between the l-KYN carboxylate group and both Arg83 and Tyr97, as a direct consequence of the reorientation of both residues. Although the l-KYN aromatic moiety is located adjacent to the flavin isoalloxazine ring as previously reported, an additional water molecule is present that is located directly above the flavin C4a/N5. This is accompanied by a slight tilting of the flavin isoalloxazine. It is interesting to note that recently published KMO class II inhibitor complexes (PDB 5NAE; 5NAG; 5NAH), where the ligand mimics both l-KYN and nicotinamide binding, reveal water molecules in a similar position directly above the C4a and C10. In each case, the flavin adopts a conformation that is tilted away from that observed in the 5NAK structure, more akin to that seen here in the KMO: l-KYN complex. In addition, the polar network between R83/Y97 and the inhibitor for one of these (5NAE) is similar to that described here for the l-KYN KMO complex (Supplementary Fig. 2). The position of the flavin isoalloxazine appears to be linked to the water molecule above C4a/N5 which, together with the adjacent water molecule located above the C10 atom, is likely to mimic the position of individual oxygen atoms in the proposed C4a peroxoflavin adduct. This suggests nucleophilic attack by the l-KYN occurs on the proximal oxygen, to generate a transient 3-oxo-l-KYN flavin adduct.

Co-crystallisation studies of *Pf*KMO with the target inhibitor compounds yielded crystal structures for 4 KMO inhibitor complexes (**1**, **4**, **9** and **13**; Supplementary Fig. 3; PDB codes 6FP1, 6FP0, 6FOY and 6FOZ). A comparison of the inhibitor **1** complex structure with the recently published class I and class II inhibitors of KMO reveals the latter to be highly similar to the class I inhibitor recently reported (PDB 5NAB; Supplementary Fig. 4)^24^. Although the aromatic moiety of **1** effectively occupies the hydrophobic binding pocket of KMO, additional space appears to be available for extending the **1** scaffold with small additional groups. A larger alkyl group at the methyl-ortho-substituent orientated away from the flavin is likely to be accommodated by the enzyme, whereas the structures of the class II inhibitors have demonstrated that extension of the methyl-ortho-substituent located in close proximity of the flavin is also possible, leading to class II type binding and flavin tilting^24^.

### Compounds 1, 4, 9 and 13 competitively inhibit KMO activity in the absence of hydrogen peroxide production

The KMO reaction is a random bi–bi mechanism proceeding through a ternary complex of KMO-bound to l-KYN and NADPH (Supplementary Figs. 5–8, steady-state enzymatic assays are described in Supplementary Methods) with a turnover value, *k*_cat_, of 8.2 ± 0.2 s^−1^ and *K*_m_ values for NADPH and l-KYN of 16.9 ± 1.5 and 12.1 ± 1.4 µM, respectively (Supplementary Fig. 6). Product inhibition studies at saturating and sub-saturating concentrations of both substrates showed mixed type inhibition patterns under all conditions (Supplementary Figs. 7 and 8), indicative of a random bi–bi reaction mechanism. This is in agreement with previous studies on other KMO enzymes^25^, and taken together with previous stopped-flow studies on the oxygen chemistry^26^ suggests an overall reaction mechanism as shown in Supplementary Fig. 5.

To validate binding modes of the four inhibitors for which crystal structures were obtained (**1**, **4**, **9** and **13**), we investigated their mechanisms of inhibition (Supplementary Figs. 9–12). Consistent with the crystal structures, each compound was found to be a competitive inhibitor of l-KYN. Inhibitor **9** is also a competitive inhibitor of NADPH, whereas compounds **1**, **4** and **13** were mixed type inhibitors with respect to NADPH. Notably, l-KYN is known to stimulate KMO reduction by NADPH and other reported KMO inhibitors mimic this effect^26^. As a consequence, these inhibitors will accumulate inflammatory hydrogen peroxide (H_2_O_2_) following oxidation of KMO-bound flavin in inhibited enzyme complexes, perhaps limiting their therapeutic potential. Notably, inhibitors **1**, **4**, **9**, **13** do not stimulate KMO reduction by NADPH in rapid mixing stopped-flow assays (Supplementary Fig. 13, Supplementary Table 1). H_2_O_2_ production was not detected with compound **1** and *Pf*KMO using a coupling reaction with o-dianisidine and horseradish peroxidase in the presence and absence of l-Kyn (Supplementary Fig. 14). This suggests that **1**, **4**, **9**, **13** inhibit KMO activity in the absence of H_2_O_2_ production and thus may be preferable for therapeutic application.

### Cheminformatics and in vitro analyses indicate that inhibitor 1 may cross the blood–brain barrier via riboflavin transporters

Standard cheminformatics^22^ were used to identify if inhibitors **1**, **4**, **9**, **13** have similarities with any endogenous metabolites and thereby the potential for transport into the brain. Using SLC-type transporters that the metabolites employ and MACCS encoding^22^, riboflavin was identified as the most similar metabolite to **1**, with a Tanimoto similarity of 0.62. Inspection of the corresponding structures revealed a common substructure of a tertiary amine linked to 4-methylbenzyl, methylene and ethylene moieties^27^. Riboflavin, however, is uncharged whereas **1** has a negative charge. Based on the similarities in chemical structure of **1** and riboflavin we surmised that **1** was the most promising candidate for transport into the brain by riboflavin transporters (SLC52 family)^28^, where SLC52A2 is expressed in almost every tissue^29^.

As a promising lead compound, we tested several in vitro pharmacokinetic parameters (further details and analytical conditions are described in Supplementary Methods) for **1**, and found that it exhibited good kinetic solubility (Supplementary Table 2) and metabolic stability in mouse hepatic microsomes (Supplementary Table 3) and rat hepatocytes (Supplementary Table 4). As an initial metric for potential brain penetrance we employed two Madin-Darby canine kidney (MDCK) cell lines—wild type (WT) and *multidrug resistance* (*MDR1*) gene expressing—to test for cell permeability. Inhibitor **1** exhibited good apparent permeability in both WT and MDR1 MDCK cells (Apical to Basolateral *P*_app_ values ~15 × 10^−6^ cm/s; Supplementary Tables 5 and 6), although the effective efflux ratio (Table 2) indicated that **1** is not a substrate for P-glycoprotein, the efflux protein encoded by *MDR1*. Notably, inhibitor **1** crossed the test membrane much more readily in the apical to basolateral direction than *vice-versa*, consistent with uptake by a transporter, complementing our observations above (Supplementary Tables 5 and 6).

**Table 2:** Effective efflux ratios for test and control compounds in MDCK cell lines

Compound	TPSA(Å^2^)	Effective efflux ratio (MDR1/wild type)		
		*n* = 1	*n* = 2	Mean
Propranolol		1.3	1.3	1.3
Vinblastine		6.7	5.2	5.95
Inhibitor **1**	67	0.64	0.84	0.74
Inhibitor **3**	71	1.7	1.7	1.7
Inhibitor **5**	71	1.5	1.4	1.45
Inhibitor **6**	37	0.81	0.43	0.62
Inhibitor **8**	67	0.65	1.3	0.975

### Inhibitor 1 ameliorates neurodegeneration in a *Drosophila* model of HD

**Fig. 3:**
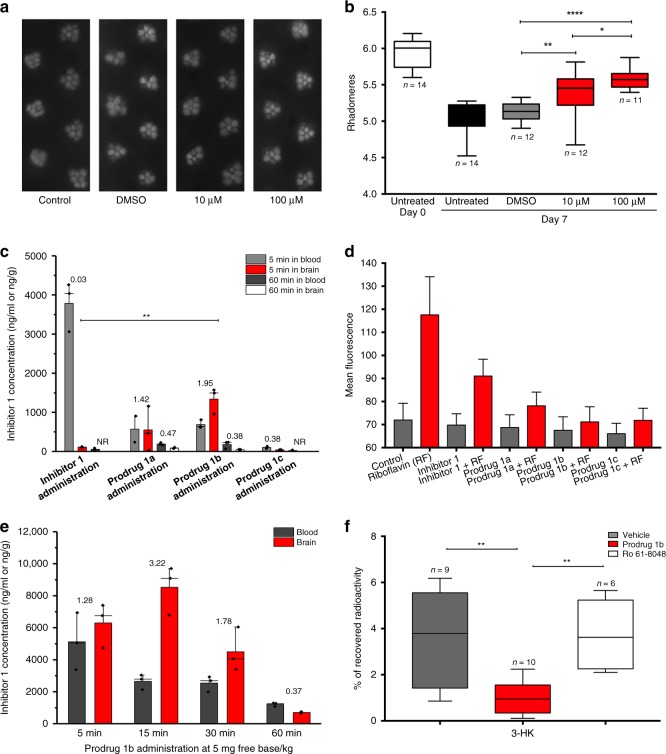
In vivo analysis of inhibitor **1** and its derivatives. **a** Representative pseudopupil images from 7 day old wild-type control flies and HD flies (treated with either vehicle (DMSO) or 10 μM/100 μM inhibitor **1**). Control flies exhibit seven visible rhabdomeres per ommatidium, whereas degeneration of these photoreceptor neurons is observed in HD flies. **b** Rhabdomere quantification of HTT93Q flies treated with inhibitor **1**. Newly enclosed HTT93Q flies were treated with 10 μM or 100  μM inhibitor **1** for 7 days. *n* = 11–14 flies per condition as labelled in the figure. *P < 0.05, **P < 0.01, ****P < 0.0001, ANOVA with Newman–Keuls post hoc test. Untreated and DMSO-treated groups served as controls. **c** The concentration of inhibitor **1** detected in the brain at 5 min and 60 min after a single intravenous administration in the mouse. The concentration of inhibitor **1** in the blood was plotted as ng/mL and in the brain was plotted as ng/g. The number above each column is the mean brain:blood ratio of inhibitor **1** detected in the sample, NR = no result as concentration below limit of detection (*n* = 3 mice, **P < 0.01, ANOVA with Tukey post hoc test). **d** The effect of inhibitor **1** and prodrugs on the fluorescence of K562 cells incubated with riboflavin (or without as a control) and read over 300 s (*n* = 300). **e** The concentration of inhibitor **1** detected in the brain and blood after single intravenous administration of prodrug **1b** in the rat (*n* = 3 rats). The number above each column is the mean brain:blood ratio of inhibitor **1** detected in the sample. **f** Percentage of [5-^3^H]-KYN that is metabolised into [^3^H]-3-HK in the striatum of awake rats 1 h after an intravenous injection of vehicle, prodrug **1b**, or Ro 61-8048. *n* = 6–10 rats as indicated in the figure panel, **P < 0.01, one-way ANOVA with Bonferroni’s multiple comparisons post hoc test. In panels **c** through **e** the values are expressed as the mean ± SEM

To test the therapeutic efficacy of **1**, we next employed a widely-used *Drosophila melanogaster* model of the inherited neurodegenerative disorder Huntington’s disease (HD)^30^, which features pan-neuronal expression of a mutant huntingtin exon 1 fragment (HTT93Q). We have previously shown that several KMO inhibitors, including UPF 648 and Ro 61-8048, are neuroprotective in this *Drosophila* model^3^. We thus tested the ability of **1** to ameliorate loss of photoreceptor neurons in the fly eye (rhabdomeres) using the pseudopupil assay. Newly enclosed HTT93Q flies treated with 10 μM or 100 μM of **1** for 7 days exhibited a significant dose-dependent reduction in neuron loss (10 μM, *P* < 0.01; 100 μM, *P* < 0.0001) when compared with untreated or vehicle (DMSO)-treated animals (Fig. 3a, b). Notably, the higher dose of **1** prevented ~57% of the neurodegeneration observed in the HD flies. Although the efficacy of **1** may not be solely due to KMO inhibition in the fly, we have previously observed similar levels of neuroprotection with other KMO inhibitors^3^.

### A prodrug strategy permits brain penetrance of inhibitor 1

Exploration of the stability and brain penetrance of **1** in mice revealed that a single intravenous bolus administration of **1** at 1 mg/kg in female C57BL/6 mice led to appreciable levels in the blood at 5 min (3785 ± 639 ng/ml), which was rapidly cleared from the blood by 60 min (Fig. 3c; Supplementary Table 7), likely a consequence of high urinary excretion (Supplementary Table 8) and secondary conjugative metabolism owing to the presence of a carboxylic acid functionality. Minimal brain penetrance of **1** was observed at either timepoint (Brain:Blood ratio 0.03). Owing to this rapid clearance and the limited brain penetrance of **1**, a number of derivatives of **1** were synthesised (**1a–h**, Table 3) (experimental details are described in Supplementary Methods). Compounds **1a–d** underwent esterification of the carboxyl group in an effort to produce prodrug variants that reduce clearance rates and release **1** upon hydrolysis. The remaining derivatives were designed to test the inhibitory potential of structurally similar amide (**1e–g**) or tetrazole (**1** **h**) bioisosteres of **1**. As expected, **1a–d** did not inhibit KMO in vitro, although **1h** demonstrated similar inhibition to **1** (Table 3). Similar to the inhibitors above, **1h** is bound in the l-KYN-binding pocket, with the tetrazole moiety in a position similar to the aniline group of l-KYN (Supplementary Fig. 3e; PDB code 6FPH). The structure of the **1h** complex is very similar to the inhibitor **1** complex, with the additional bulk of the triazole group accommodated for by reorientation of R83. This allows a very similar network of polar contacts between the protein and **1h** to be formed.

**Table 3:**
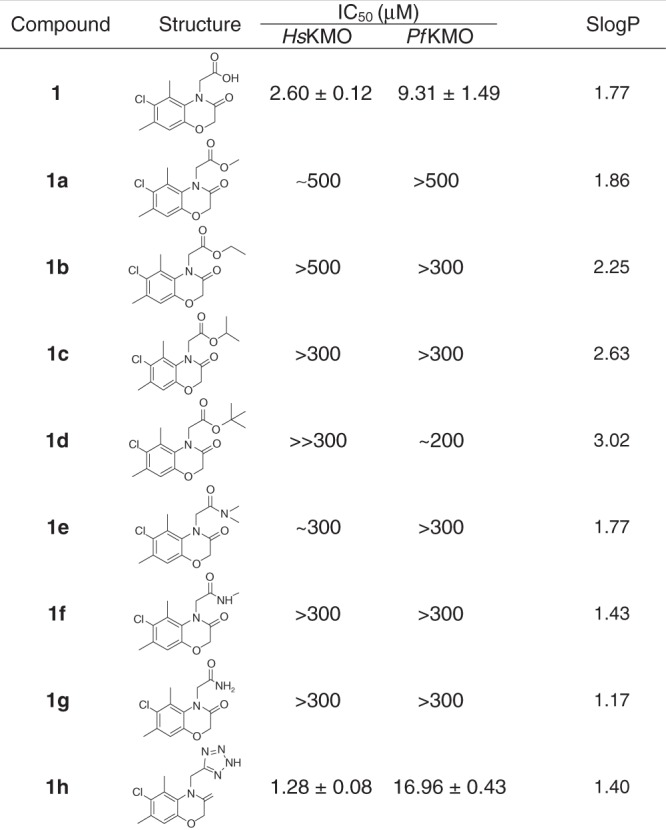
Structures, IC_50_ values and calculated SlogP values for compound **1** and derivatives

Slog*P* values calculated using KNIME/RDKit using implementation described^33^

Pharmacokinetic profiles of the **1** prodrugs (**1a–d**) were explored after intravenous bolus administration of 1 mg/kg. **1a**, **1b** and **1c** all released **1** in both the blood and brain, whereas **1** was undetectable in **1d**-treated animals (Fig. 3c; Supplementary Table 7). Notably, brain levels of **1** derived from prodrugs **1a** and **1b** were higher than in the blood 5 min after treatment, with Brain:Blood ratio of 1.42 and 1.95, respectively. The brain levels of **1** derived from these compounds dropped markedly 60 min after treatment, which may indicate efflux of **1a** and **1b** from the CNS. The isostere **1h** exhibited very limited brain penetrance (Supplementary Table 7) and blood levels were in fact ~50% lower than those observed for **1**, suggesting that this isostere is cleared even more rapidly from the blood. Inhibitor **1** as well as prodrugs **1a, 1b** and **1c** inhibit riboflavin uptake by K562 cells when incubated with riboflavin, consistent with the cheminformatic prediction of uptake by the riboflavin transporter (Fig. 3d; Supplementary Fig. 15). Furthermore, **1b** showed higher inhibition of riboflavin uptake compared with **1** and the other prodrugs.

We next extended our pharmacokinetic analyses by testing the stability and brain penetrance of **1b** and released **1** in Wistar rats (further details are described in Supplementary Methods). In all, 5 mg/kg of **1b** was administered intravenously, and levels of **1b** and **1** were measured in the blood and brain at several timepoints post-administration (5, 15, 30 and 60 min) (Supplementary Tables 9 and 10). Supporting our data in mice, we found compound **1** derived from **1b** in the brain at all timepoints, with a maximal Brain:Blood ratio of 3.22 at 15 min (Figure 3e). **1b** was also detected in the brain, although maximal concentration was 5 min post administration, with very low levels of unbound compound detected in this tissue (**1b** brain Fu = 0.016) (Supplementary Table 9). Notably, the unbound brain concentrations of **1** (**1** brain Fu = 0.30) released from **1b** throughout the timecourse were ~2–20-fold higher than the compound **1** IC_50_ for KMO inhibition in brain homogenates from Sprague–Dawley rats determined via in vitro assays (Supplementary Fig. 16), indicating that sufficient levels of **1** should be present in the brain to affect KMO activity.

### Peripheral administration of prodrug 1b decreases de novo synthesis of 3-HK in the CNS

Having found that appreciable levels of inhibitor **1** are released in the CNS following the intravenous administration of the prodrug **1b**, we next assessed if this pharmacological intervention modulates de novo KP metabolism in the brain in vivo, using a radioactive tracer approach with [^3^H]-KYN, which leads to the production of newly formed [^3^H]-KYNA, [^3^H]-3-HK and [^3^H]-QUIN^31^. The prodrug (5 mg/kg) was administered intravenously to rats, immediately followed by an intrastriatal infusion of [^3^H]-KYN. Tritiated KP metabolites were analysed after 1 h. A significant ~70% reduction (*P* < 0.01) in [^3^H]-3-HK was observed (Fig. 3f) in parallel with minor increases in [^3^H]-QUIN and [^3^H]-3-KYNA (data not shown)—mirroring the results using a focal intracerebral injection of a KMO inhibitor^17^. In contrast, no reduction of [^3^H]-3-HK was detected in rats receiving an intravenous infusion of Ro 61-8048 (50 mg/kg), a KMO inhibitor that does not cross the blood–brain barrier^4,12^. Taken together, these data suggest that compound **1** released locally by the prodrug **1b** effectively inhibits KMO activity in the CNS.

## Discussion

The therapeutic potential of targeting KMO in neurodegenerative diseases is supported by several studies published over the past ~15 years using genetic and pharmacological approaches. Indeed, a flurry of recent activity has led to the generation and characterisation of a number of novel, potent KMO inhibitors, which are efficacious in a variety of disease models^4,7,13,14,26^. However, a limitation of these compounds when considering neurodegenerative disorders is that none appreciably penetrate the blood–brain barrier to enter the brain. While inhibition of KMO in the blood is likely sufficient to increase CNS levels of neuroprotective KYNA via increased transport of l-KYN to the brain^4^, KMO inhibition in the brain would have the added benefit by decreasing levels of the downstream neurotoxic metabolites. Thus the development of brain-penetrant KMO inhibitors is of great interest to the neurodegeneration research community, and holds the promise of delivering particularly robust neuroprotection. Herein, we present the first characterisation of a brain-penetrant KMO inhibitor.

Our studies support the notion that **1** is a neuroprotective KMO inhibitor that can be delivered to the brain using a prodrug strategy, as exemplified by **1b**. We show that this approach can lead to inhibition of KMO in the brain, thereby dramatically decreasing de novo 3-HK synthesis in the CNS. This work will form the basis for future studies, detailing the pharmacokinetics and pharmacodynamics of these compounds, and testing of their therapeutic potential. The hope is that this will lead to clinical therapies for neurodegenerative disease.

## Methods

### Screening of potential KMO inhibitors

Small-molecule compounds were supplied by Biofocus (Charles River Laboratories International Inc., MA, USA), which were identified in silico as potential inhibitors (further details are described in the Supplementary Methods). Initial inhibition assays were performed against *Pseudomonas fluorescens* KMO (*Pf*KMO) and *Homo sapiens* KMO (*Hs*KMO) by monitoring the time-dependent absorbance change of NADPH at 340 nm. Purified KMO protein (200 nM) was incubated in 100 µL reaction buffer (20 mM potassium phosphate pH 8.0, 7 mM 2-mercaptoethanol) containing 100 μM NADPH, 100 μM l-Kyn and 20 μM potential inhibitors in 96-well plates. The change in absorbance at 340 nm was measured using a Synergy™ HT microplate reader (BioTek, VT, USA) for 25 min at 37 °C. The inhibitors identified in the initial screens were validated and re-assayed individually in a 1 cm path-length quartz micro cuvette. IC_50_ values of potential compounds towards *Pf*KMO or *Hs*KMO <200 μM were considered as indicative of KMO inhibition. Suppliers for target inhibitor compounds are listed in Supplementary Table 11.

### Expression and purification of KMO

The codon optimised gene for *P. fluorescens* KMO (accession number: Q84HF5) containing mutations of two cysteine residues (252 and 461) to serine was synthesised (GeneArt, ThermoFisher), sub-cloned into pET17b and transformed into *E. coli* BL21 (DE3) competent cells for expression. Protein was expressed by growing transformed cells in auto induction LB medium (Formedium^TM^, glucose/lactose ratio 1:4) containing 100 µg/mL ampicillin for 24 h at 22 °C. Cells were harvested by centrifugation at 6000 g for 15 min at 4 °C, resuspended in lysis buffer (20 mM HEPES pH 7.5, 10 mM NaCl, 1 mM DTT) supplemented with protease inhibitor cocktail and lysed by sonication (15 × 15 s). The cell lysate was centrifuged at 180,000 g for 1 h at 4 °C to remove cell debris. The soluble cell lysate was loaded onto a  Q-sepharose column equilibrated with anion exchange buffer (20 mM HEPES pH 7.5, 1 mM DTT with 10 mM NaCl) and bound protein was eluted with a gradient (five column volumes) of increasing NaCl concentration from 50 to 100 mM in anion exchange buffer. Fractions containing KMO were pooled and precipitated in 50% saturated ammonium sulphate by adding precipitation buffer (20 mM HEPES pH 7.5, 3 M ammonium sulphate, 1 mM DTT). The precipitated protein was pelleted by centrifugation at 12,000 g for 20 min, resuspended in a small volume of size exclusion buffer (20 mM HEPES pH 7.0, 150 mM sodium acetate, 1 mM DTT) and passed down a Superdex 75 column. Pure *Pf*KMO was pooled and stored at −80 °C. *Homo sapiens* KMO (*Hs*KMO) was expressed and purified as previously described^23^. Cell pellets were thawed at room temperature and resuspended in Lysis/Solubilisation buffer (20 mM potassium phosphate buffer pH 7.5, 10% glycerol, 1% n-dodecyl b-d-maltoside (DDM), 300 mM NaCl, 7 mM 2-mercaptoethanol, 1 mM EDTA and 50 μM FAD-containing protease inhibitors and Benzonase (10,000 dilution). Cells were lysed by sonication on ice and cell debris was removed by centrifugation at 15,000 rpm for 30 min. Soluble lysate was incubated with pre-equilibrated glutathione uniflow resin (Clontech). The resin was then packed and washed with washing buffer (20 mM potassium phosphate buffer pH 7.5, 10% glycerol, 0.015% DDM, 150 mM NaCl, 7 mM 2-mercaptoethanol, 1 mM EDTA, 1 mM PMSF, 50 μM FAD). 0.5 mL fractions were eluted with elution buffer (washing buffer supplemented with 20 mM reduced glutathione, pH 7.0) and fractions containing GST-KMO were pooled, concentrated and loaded onto a Superdex 200 (10/30) size exclusion chromatography column (GE Healthcare). The GST part of the fusion protein was cleaved from *Hs*KMO by overnight incubation and gentle shaking with thrombin (1 unit/100 μg of protein) before size exclusion chromatography. Pure GST-*Hs*KMO or cleaved *Hs*KMO were pooled and stored at −80 °C. The purity of final fractions was analysed by SDS–PAGE.

### Crystallisation of *Pf*KMO

To obtain *Pf*KMO structures in complex with substrate or inhibitor purified protein in 20 mM HEPES pH 7.0, 20 mM sodium acetate, 1 mM DTT was incubated with each small molecule (1 mM) overnight at 4 °C on a roller shaker. The solution was clarified by centrifugation prior to concentrating to a final protein concentration of 12–15 mg/mL. Crystals of KMO-ligand complexes were obtained by mixing 200 nL of protein with 200 nL of D5 condition (0.2 M sodium acetate trihydrate, 0.1 M sodium cacodylate pH 6.5, 18% w/v PEG 8000) in SG1 screens (Molecular Dimensions Ltd., Newmarket, UK). Crystals were obtained by incubation at 4 °C for 72 h using the sitting drop vapour diffusion technique with microseeding from KMO crystals generated previously.

Data were collected from single cryo protected crystals of *Pf*KMO at beamlines i03, i04 and i04-1 (Diamond Light Source). All data were indexed, scaled and subsequently integrated with Xia2. Structure determination was initially performed by molecular replacement in Phaser using a search model derived from the previously solved *Saccharomyces cerevisiae* KMO (4J33). A combination of automated and manual rebuilding and refinement in Phenix and COOT were used to produce each of the refined models. Validation with both Molprobity and PDB_REDO were integrated into the iterative rebuild process. Complete data collection and refinement statistics are available in Supplementary Table 12.

### Stopped-flow spectrophotometry

The reduction rate of the flavin cofactor by NADPH was measured using a stopped-flow spectrophotometer (Applied Photophysics Ltd), which was housed in an anaerobic glovebox (Belle Technology). Anaerobic mixtures of 20 μM *Pf*KMO in the presence and absence of saturating concentrations of l-KYN (500 µM) or inhibitor (more than 100 times of *K*_i_ according to Supplementary Table 13, **1**: 120 µM, **4**: 700 µM, **9**: 60 µM, **13**: 6 µM, **1** **h**: 220 µM) were mixed against anaerobic NADPH (4 mM) at 25 °C. The reduction of the flavin was monitored by the decrease in the absorbance of the flavin at 450 nm and transients were fitted to either double or triple exponentials.

### Hydrogen peroxide production

H_2_O_2_ production rates from *Pf*KMO inhibitor complexes were performed using a horseradish peroxidase (HRP) assay. NADPH consumption rate was measured in the presence and absence of an excess of KMO inhibitors (400 μM). For determination of H_2_O_2_ production rates, *Pf*KMO (0.1 μM) was incubated with 100 μM NADPH, 200 μM l-KYN, 400 μM o-dianisidine and ∼5 units HRP in the presence and absence of excess KMO inhibitors. The rate of oxidation of o-dianisidine by H_2_O_2_, catalysed by HRP, was monitored at 440 nm (Δε 440 nm = 11.3 mM^−1^ cm^−1^). To eliminate the l-Kyn competition binding effect, the H_2_O_2_ production rates of KMO inhibitors were also measured without l-Kyn with the same method.

### Mouse brain penetration studies

Inhibitor **1** (and prodrugs **1a**, **1b**, **1c** and **1d**) was dosed as a bolus solution intravenously at 1 mg free base/kg (dose volume: 5 mL/kg; dose vehicle: 5% DMSO; 95% saline (inhibitor **1**) or 5% DMSO; 40% PEG400; 55% MilliQ H_2_O (prodrugs **1a**, **1b**, **1c** and **1d**)) to female C57BL6 mice (*n* = 6). At 5 min and 60 min following intravenous bolus injection of test compound, mice (*n* = 3/timepoint) were placed under terminal anaesthesia with isofluorane. A blood sample was taken by cardiac puncture, the mice decapitated and the brain removed. Brain weight was noted and each brain homogenised with two volumes of methanol (2 mL/g brain). Each blood sample was diluted with distilled water (1:10). Diluted blood and the brain homogenates were then stored frozen until preparation and analysis by UPLC-MS/MS. For each mouse at each timepoint for **1**, the concentration in brain (ng/g) was divided by the concentration in blood (ng/mL) to give a brain:blood ratio. The mean value obtained was quoted. For prodrugs **1a** to **1d**, only the concentration of **1** in blood (ng/mL) and brain (ng/g) was reported.

### Rat brain penetration studies

Prodrug **1b** was dosed as a bolus solution intravenously at 5 mg free base/kg (dose volume: 1 mL/kg; dose vehicle: 10% DMSO; 60% PEG400; 30% MilliQ H_2_O) to male Han Wistar rats (*n* = 12). At 5, 15, 30 and 60 min following intravenous bolus injection of prodrug **1b**, mice (*n* = 3/timepoint) were placed under terminal anaesthesia with isofluorane. A blood sample was taken by cardiac puncture, the mice decapitated and the brain removed. Brain weight was noted and each brain homogenised with two volumes of methanol:water (1:1; 2 mL/g brain). Each blood sample was diluted with distilled water (1:2). Diluted blood and the brain homogenates were then stored frozen until preparation and analysis by UPLC-MS/MS, monitoring for prodrug **1b** and production of inhibitor **1**. For each rat at each timepoint for **1**, the concentration in brain (ng/g) was divided by the concentration in blood (ng/mL) to give a brain:blood ratio. The mean value obtained was quoted.

### Bioanalysis by ultra-performance chromatography and tandem mass spectrometry

A rapid and sensitive ultra-performance chromatography/tandem mass spectrometry (UPLC-MS/MS) method was developed and validated for the determination of inhibitor **1** and prodrug **1b** (rat only). Chromatographic analysis was conducted on a Waters Acquity™ UPLC system. Chromatographic separation was performed with an Acquity™ UPLC BEH C18 column (50 × 2.1 mm, 1.7 µm, 130Å; Waters Corp.) maintained at 40 °C in a column oven. The mobile phase consisted of water with 0.01% formic acid and ACN with 0.01 % formic acid and it was delivered at a flow rate of 0.6 mL/min under gradient eluent conditions within an analysis cycle time of 3 min. The mass detection was conducted on a Waters Xevo™ TQs mass spectrometer equipped with a ScanWave™ technology. Quantitation of the analyte was performed in electrospray positive ionisation mode (ESI^+^) using multiple reaction monitoring. The optimised fragmentation transition was *m/z* = 270.17 → 196.19 (mouse) or *m/z* = 270.17 → 223.76 (rat) for inhibitor **1** and *m/z* = 297.83 → 195.74 for prodrug **1b** (rat).

Sample extraction for blood and brain homogenate was by protein precipitation (1:3 or 1:6) using ACN containing suitable internal standard. Extracts were vortexed followed by centrifugation (2800 rpm for 10 min). An aliquot of each supernatant was then diluted two-fold in distilled water ready for analysis by UPLC-MS/MS.

The weighted 1/*x*^2^ calibration line was linear in the dynamic range of 1–5000 ng/mL for inhibitor **1** and 1–1000 ng/mL for prodrug **1b** with a correlation coefficient (*r*^2^) > 0.99 (inhibitor **1**) or 0.98 (prodrug **1b**). The quality control samples (QC) were prepared by using the same spiking scheme as the calibration samples and up to four different target concentration brain levels: 2 ng/mL (LQC), 40 ng/mL (MQC), 100 ng/mL (HQC) and 2000 ng/mL. Data collection, peak integration and calculations were performed using MassLynx software, version 2.1.

### Therapeutic efficacy of inhibitor 1 in *Drosophila melanogaster* model

Flies were kept on standard maize food at 25 °C with a 12:12 light/dark cycle. HTT93Q exon 1 expressing flies were generated by crossing female virgins carrying the *UASHTT93Qexon1* transgene^30^ (a gift of Larry Marsh and Leslie Thompson, University of California, Irvine) to males of *elavGAL4* [c155] driver line, obtained from the Bloomington *Drosophila* Stock Center. For the drug feeding experiments, inhibitor **1** was dissolved in dimethylsulfoxide (DMSO) and added to maize media at the experimental concentrations. Newly emerged HTT93Q exon 1 flies were transferred to media supplemented with DMSO or drug and changed daily to fresh media for the duration of the experiment. At day 7, rhabdomeres were scored as previously described^5^. In brief, flies were anaesthetised with CO_2_, their heads removed and then mounted on microscope slides using nail polish. Rhabdomeres were examined using a Nikon Optiphot-2 light microscope, with ~ 80 ommatidia scored per fly.

### Intrastriatal injection of [5-^3^H]-kynurenine

The radioactive tracer method previously described by Guidetti et al. (1995)^31^ was used to study acute KP metabolism in the brain in vivo. To this end, adult male Sprague–Dawley rats (weighing 270–300 g) were anesthetized with isoflurane using a vaporiser (induction dose 5%, maintenance dose 2%, v/v, in 100% oxygen) and placed in a stereotaxic frame. A guide cannula was positioned unilaterally over the striatum (AP: 0.8 mm anterior to bregma, ML: ±2.7 mm from midline, DV: 4.5 mm below dura; hemispheres counter-balanced) and secured to the skull with acrylic dental cement. On the next day, rats were injected intravenously with either vehicle or prodrug **1b** (5 mg/kg), then immediately rats were infused intrastriatally with 6 µL of [^3^H]-kynurenine (2.5 µCi) over 10 min using a microinfusion pump. To assess the effect of KMO inhibition in the striatum, all animals were killed 1 h after the start of the [^3^H]-kynurenine infusion, and their brain was quickly removed. The striata were rapidly dissected out on ice, frozen on dry ice and stored at 80 °C until processed. After thawing, tritiated KP metabolites were detected and analysed as described previously^31^. The production of newly formed [^3^H]-KYNA, [^3^H]-3-HK and [^3^H]-QUIN was expressed as a percentage of the total radioactivity recovered per striatum. HPLC conditions: 100 mM ammonium phosphate (11.5 g/L)**;** 100 mM glacial acetic acid (5.75 mL/L = 6.03 g/L); 1.5 mM 1-octanesulfonic acid (0.34 g/L); 4% ACN pH 3.2 with 1:1 H_3_PO_4_/acetic acid. The mobile phase was flown at 1.2 ml/min (Waters 515 HPLC Pump) through YMC-Pack Pro C18 (250 × 4.6 mm) column and detected with UV (Applied Biosystems 785A Absorbance Detector) at 254 nm then mixed with LabLogic FlowLogic U scintillation fluid flowing at 2.7 mL/min and radiation was detected (Berthold Technologies FlowStar LB514).

### Competition assays for the uptake of riboflavin and KMO inhibitors

Riboflavin transporters are equilibrative rather than concentrative, and the uptake of the inhibitors would be hard to measure directly. To assess whether the KMO inhibitors were substrates for riboflavin transporters, we assessed their ability to compete with riboflavin for uptake into cells. Riboflavin is intensely fluorescent, and is taken up in human cells by three main transporters SLC52A1, SLC52A2 and SLC52A3^28^. SLC52A2 is the most highly expressed, but each has a *K*_m_ for riboflavin of ~1 μM. Consequently, we incubated cells with 1 μM riboflavin in the absence and presence of the various KMO inhibitors. There was a background fluorescence of some 100 relative fluorescence units. Cellular uptake was quite heterogeneous, but Supplementary Fig. 13 shows that for each of the three inhibitors illustrated, there is an inhibition of uptake. The data are summarised in Fig. 3c, and are consistent with the view that at least some of the KMO inhibitor uptake is likely to go via riboflavin transporters.

### Statistics and reproducibility

Statistical analyses were performed using Prism 7 (GraphPad Software Inc). ANOVA with Newman–Keuls post hoc tests were used for analyses of rhabdomere degeneration, with *P* values < 0.05 considered significant for the comparisons. For analysis of brain penetration of compounds, ANOVA with Tukey post hoc tests were used and *P* values < 0.01 were considered significant. For assessment of de novo 3-HK synthesis via [5-^3^H]-kynurenine, ANOVA with Bonferroni’s multiple comparisons post hoc tests were employed with *P* values < 0.01 considered significant. All the measurements were done with at least three repeats. However, owing to sensitivity limitations in mass spectrometry detection, two measurements of brain penetration were restricted to only two replicates. All the values are expressed as the mean ± SEM or mean ± SD.

### Ethical regulations

The pharmacokinetics studies were performed at the Drug Discovery Unit, School of Life Sciences, University of Dundee, UK. All regulated procedures on living animals in the DDU were carried out under the authority of a project licence issued by the Home Office under the Animals (Scientific Procedures) Act 1986, as amended in 2012 (and in compliance with EU Directive EU/2010/63). Licence applications were approved by the University’s Ethical Review Committee (ERC) before submission to the Home Office. The ERC has a general remit to develop and oversee policy on all aspects of the use of animals on University premises and is a sub-committee of the University Court, its highest governing body. For the mouse studies female C57Bl6 mice were employed (6–12 weeks old). For the rat studies male Han Wistar rats were employed (7–9 weeks old).

Analysis of de novo 3-HK synthesis was performed at the Maryland Psychiatric Research Center, University of Maryland, USA. Animals were housed in a temperature-controlled, AAALAC-approved animal facility on a 12/12h-light/dark cycle with unlimited access to food and water. The experimental protocol was approved by the Institutional Animal Care and Use Committee of the University of Maryland School of Medicine. Male, Sprague–Dawley rats were employed (10-week old).

### Reporting summary

Further information on research design is available in the Nature Research Reporting Summary linked to this article.

## Supplementary information

Supplementary Information

Description of Additional Supplementary Files

Reporting Summary

Supplementary Data

## Data Availability

The atomic coordinates and experimental data (codes 6FP0, 6FP1, 6FPH, 6FOX, 6FOY & 6FOZ) have been deposited in the Protein Data Bank (www.wwpdb.org). Raw data used for Fig. 3 are available in Supplementary Data 1. All other data are available from the corresponding author on reasonable request.
