# Supplementary material for: A brain-permeable inhibitor of the neurodegenerative disease target kynurenine 3-monooxygenase prevents accumulation of neurotoxic metabolites
Source: Commun Biol. 2019 Jul 24;2:271. doi: 10.1038/s42003-019-0520-5 (PMC6656724; doi:10.1038/s42003-019-0520-5)
Supplement: Supplementary file 1 — Supplementary Information [file 42003_2019_520_MOESM1_ESM.pdf]

1. Supplementary Figures

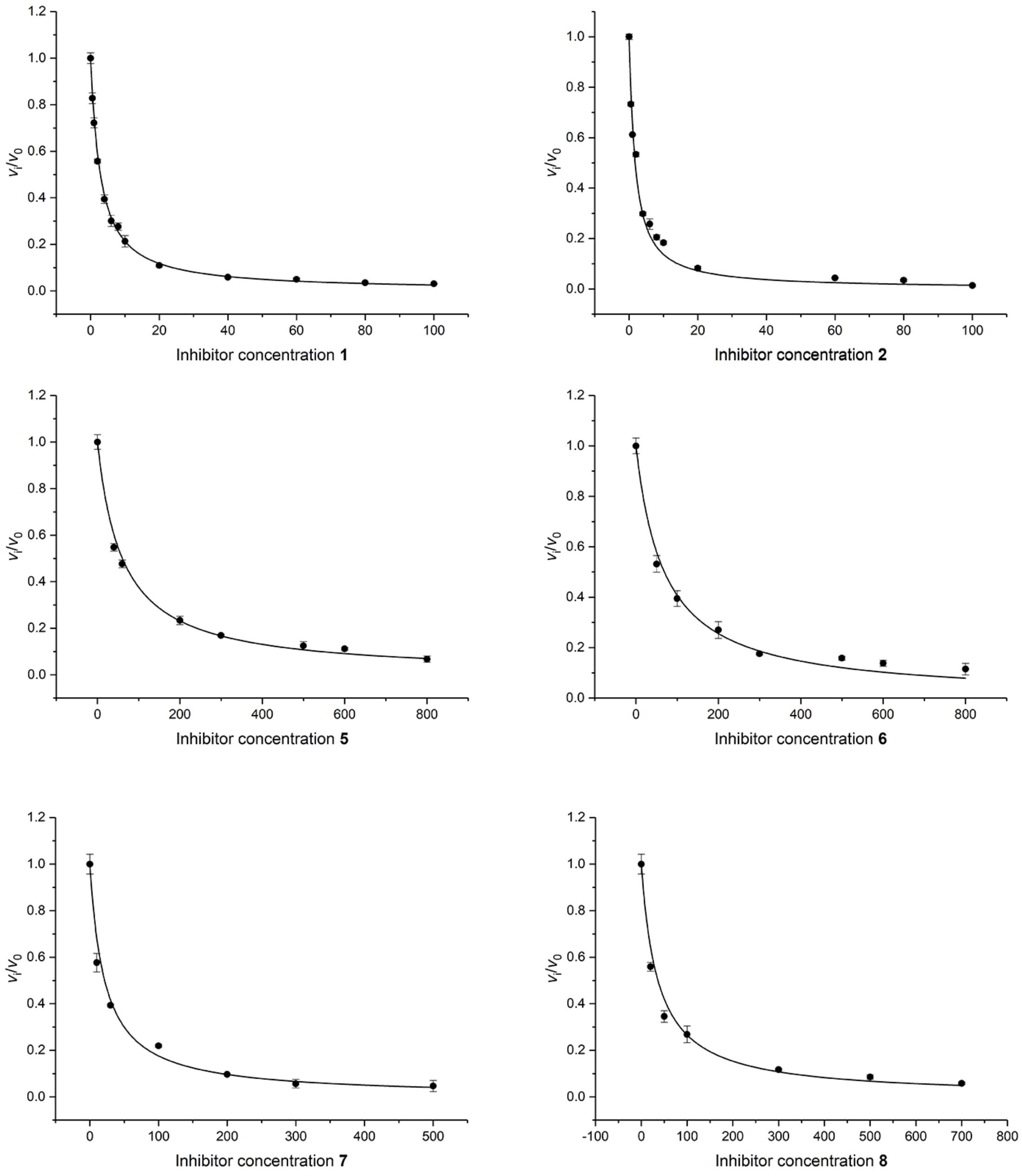

Supplementary Figure 1. Examples of inhibition data used to generate IC_{50} values for selected KMO inhibitors. Fixed concentration of L-KYN (200µM) and NADPH (100µM) were added into reaction buffer (*Hs*KMO: 20mM potassium phosphate pH 8.0, 7 mM 2-mercaptoethanol; *Pf*KMO: 20 mM HEPES, 10mM sodium acetate, pH 7.5, 2mM DTT). The reaction was initiated by the addition of KMO enzyme (*Hs*KMO: 0.5µM; *Pf*KMO: 0.1 µM). KMO activity was measured in the presence of a range of inhibitor concentrations.

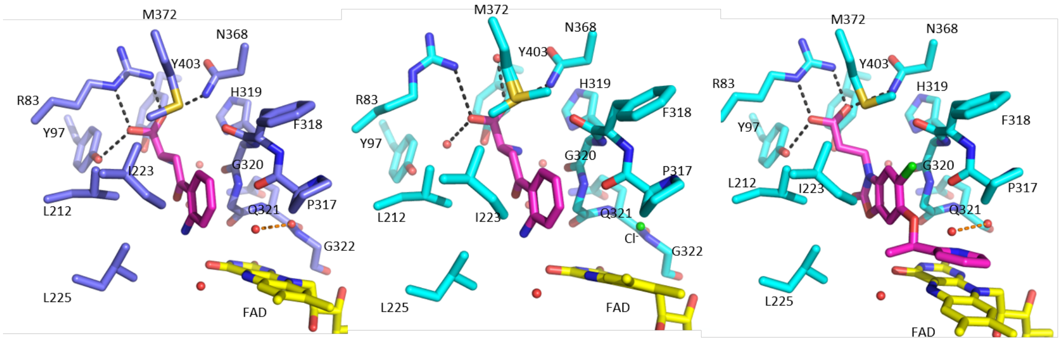

Supplementary Figure 2. Structure of the *pf*KMO L-KYN complex. Panels A, B and C depict a side-by-side comparison of the KMO L-KYN complex. (A); KMO L-KYN complex reported here, (B); the recently published KMO L-KYN complex (PDB 5NAK), (C); one of the recently published class II KMO inhibitor complexes. Key amino acids are shown in atom coloured sticks, and hydrogen bonding contacts formed with the ligand carboxylate group are shown as black dotted lines. The two water molecules present in (A) and (C) that likely mimic the position of oxygen atoms in the peroxo-flavin species are connected by an orange dotted line.

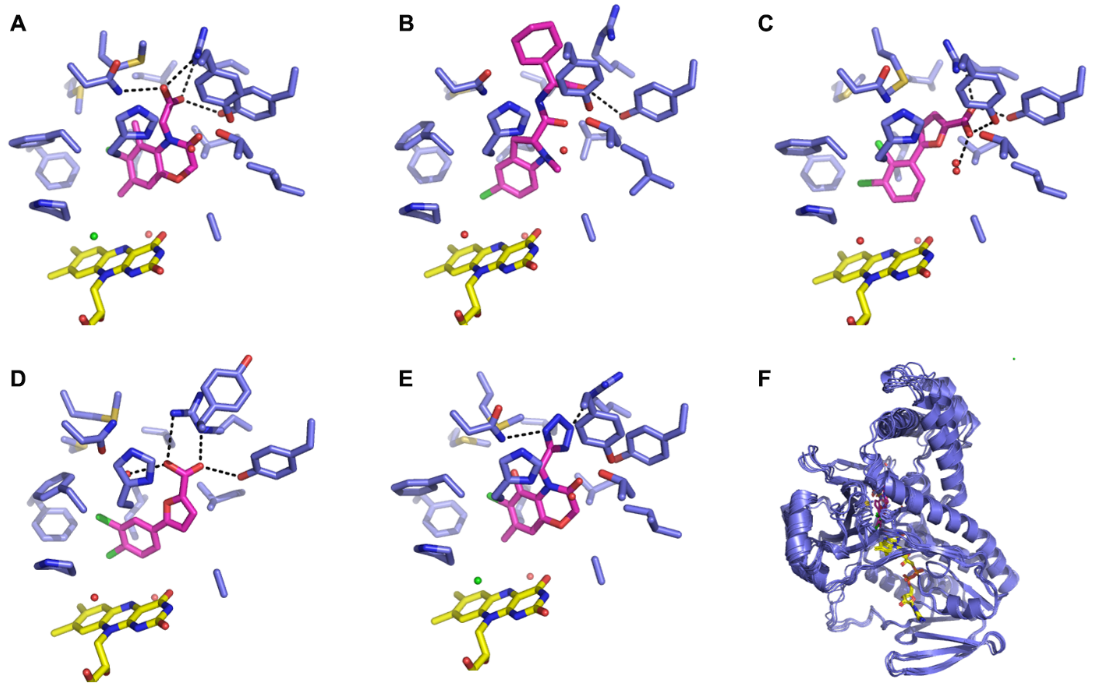

Supplementary Figure 3. Structure of *pf*KMO inhibitor complexes. Panels A-E depict a Side-by-side comparison of the KMO inhibitor complexes for **1** (A), **4** (B), **9**, (C), **13** (D) and **1h** (E). Key amino acids are shown in atom coloured sticks, and hydrogen bonding contacts formed with the ligand carboxylate group (or tetrazole for compound 1H in Figure S3E) are shown as black dotted lines. Panel F depicts a cartoon overlay of the corresponding 5 KMO structures (only one monomer is shown), revealing that large changes occur in the substrate binding regions as a consequence of the distinct shape of the respective inhibitor compounds.

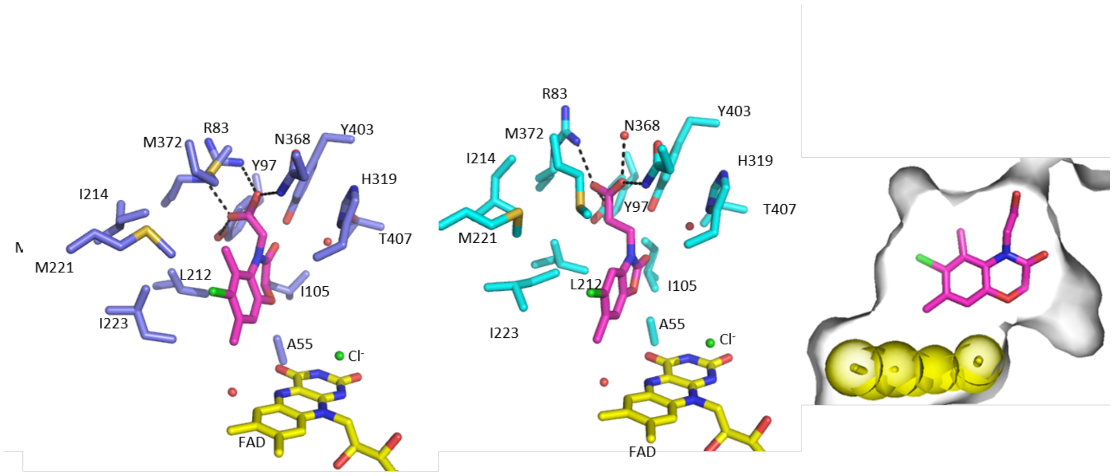

Supplementary Figure 4. Compound 1 is a class I inhibitor. Panels A and B depict a side-by-side comparison of the *Pf*KMO - compound **1** complex and the recently reported class I inhibitor complex (PDB code 5NAB). Key amino acids are shown in atom coloured sticks, and hydrogen bonding contacts formed with the ligand carboxylate group are shown as black dotted lines. Panel C depicts a cross section through the active site of the KMO - compound **1** complex, with the solvent accessible surface of the protein shown in grey. This suggests both the *ortho*-methyl groups can likely be replaced with larger substituents to further improve on inhibitor properties.

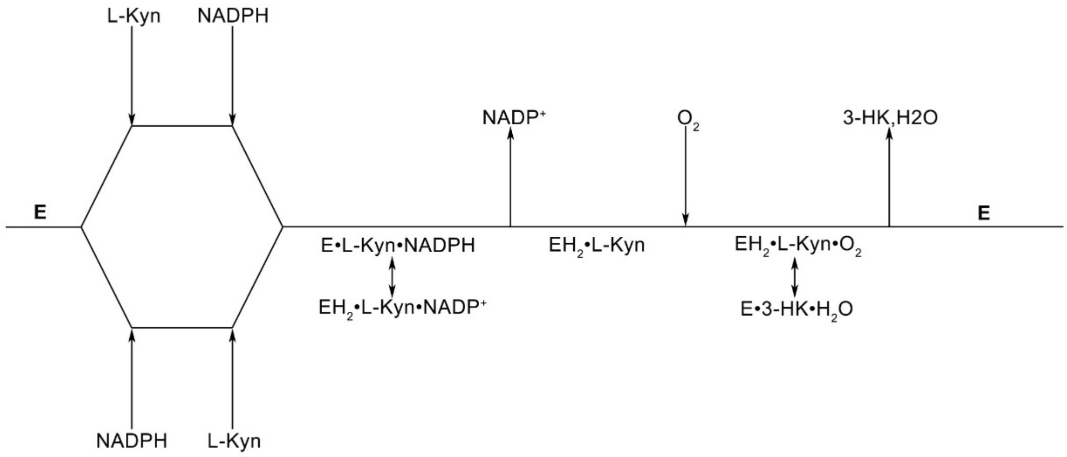

Supplementary Figure 5. Overall KMO reaction mechanism in the shorthand notation of Cleland[1].

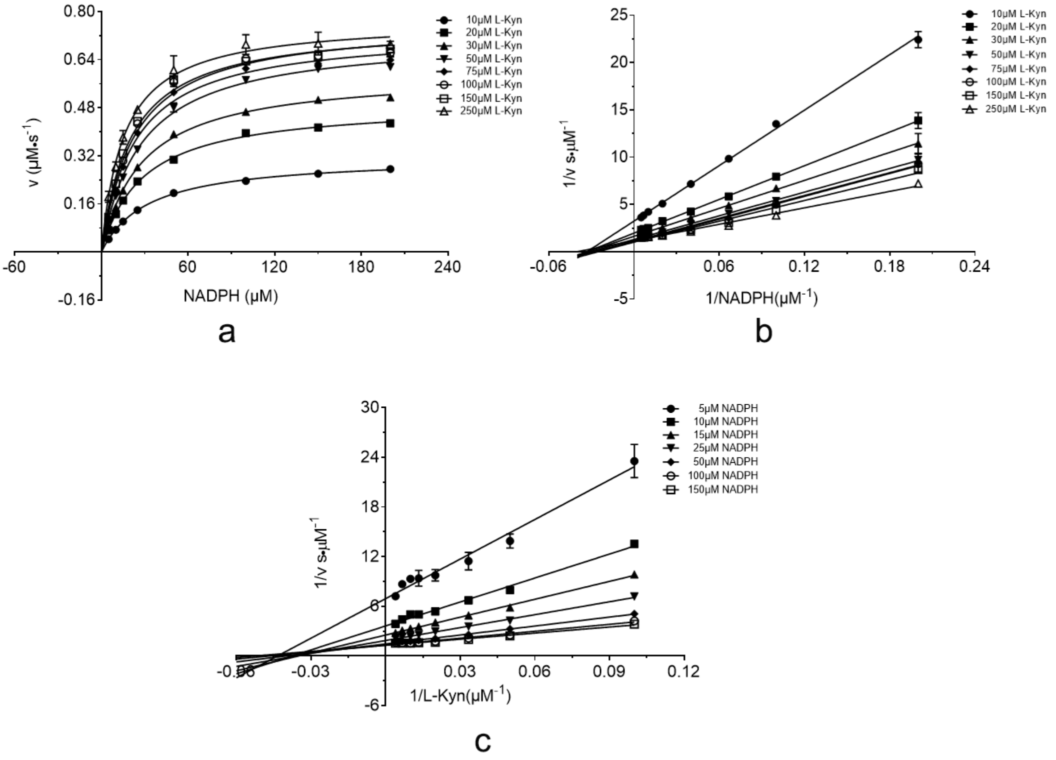

Supplementary Figure 6. Steady state kinetics study of *Pf*KMO. a) The steady-state rate measured at varying concentrations of NADPH (5, 10, 15, 25, 50, 100, 150, and 200 µM) and L-KYN (10, 20, 30, 50, 75, 100, 150, and 250 µM). b) Double reciprocal plot of 1/velocity vs. 1/[NADPH] at fixed concentrations of L-KYN (10, 20, 30, 50, 75, 100, 150, 200 µM). c) Double reciprocal plot of 1/velocity vs. 1/[L-KYN] at fixed concentrations of NADPH (5, 10,15, 25, 50, 100, 150 µM).

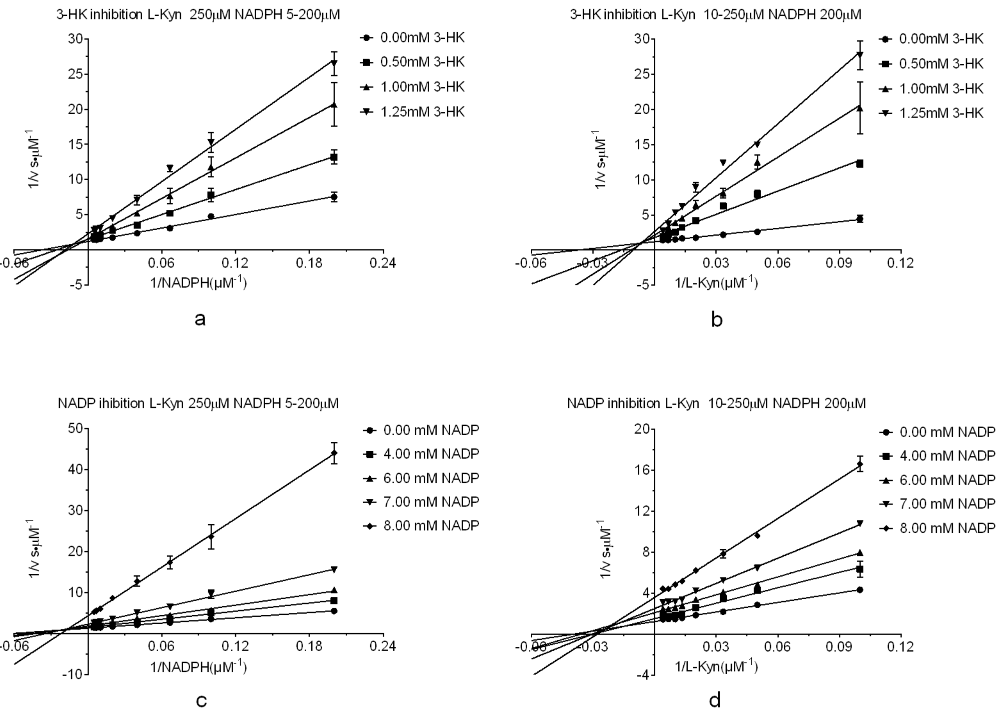

Supplementary Figure 7. Product inhibition of *Pf*KMO activity at saturating concentrations of the L-KYN and NADPH substrates. Data are plotted as double reciprocal plots of 1/velocity vs the following: a) 1/[NADPH] at 250 μM L-KYN and increasing concentrations of 3-HK; b) 1/[L-KYN] at 200 μM NADPH and increasing concentrations of 3-HK; c) 1/[NADPH] at 250 μM L-KYN and increasing concentrations of $NADP^+$; d) 1/[L-KYN] at 200 μM NADPH and increasing concentrations of $NADP^+$.

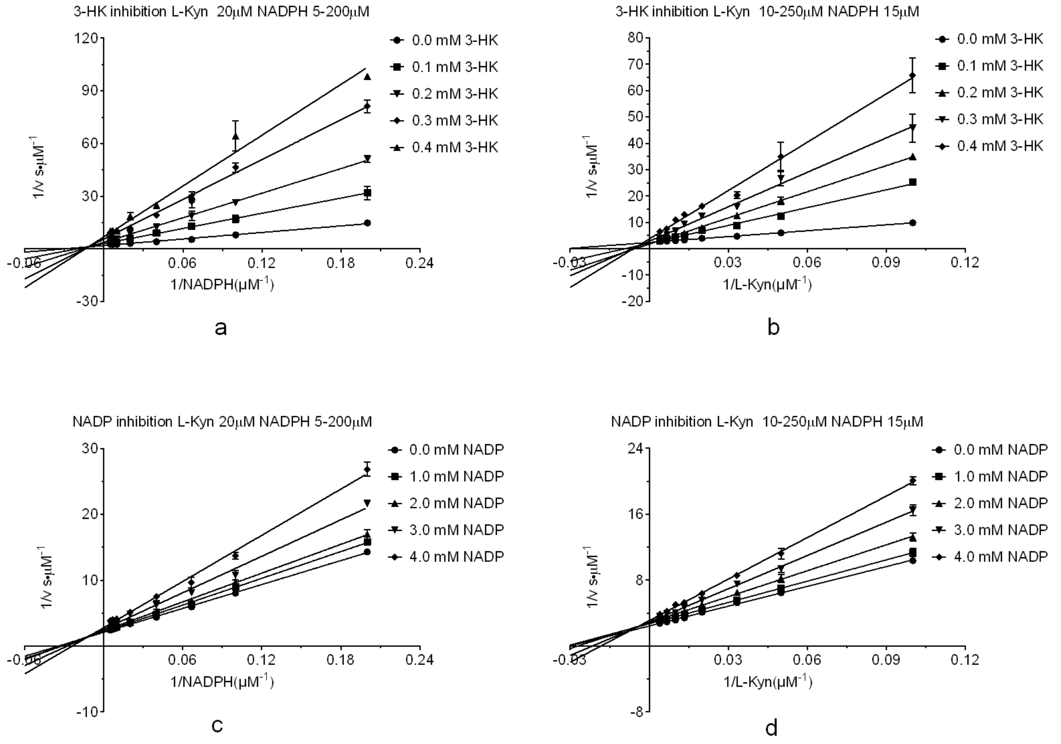

Supplementary Figure 8. Product inhibition of *Pf*KMO activity at sub-saturating concentrations of the L-KYN and NADPH substrates. Data are plotted as double reciprocal plots of 1/velocity vs the following: a) 1/[NADPH] at 20 μM L-KYN and increasing concentrations of 3-HK; b) 1/[L-KYN] at 15 μM NADPH and increasing concentrations of 3-HK; c) 1/[NADPH] at 200 μM L-KYN and increasing concentrations of $NADP^+$; d) 1/[L-KYN] at 15 μM NADPH and increasing concentrations of $NADP^+$.

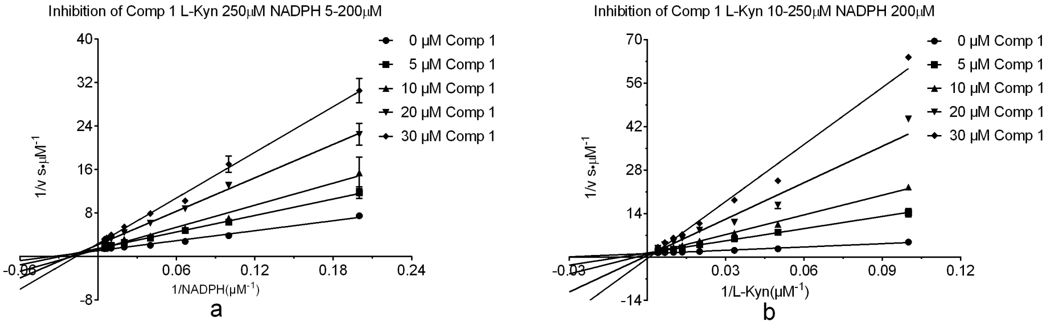

Supplementary Figure 9. Inhibition of *Pf*KMO activity by compound 1 at saturating concentrations of the L-KYN and NADPH substrates. Data are plotted as double reciprocal plots of 1/velocity vs the following: a) 1/[NADPH] at 250 μM L-KYN and increasing concentrations of compound 1; b) 1/[L-KYN] at 200 μM NADPH and increasing concentrations of compound 1.

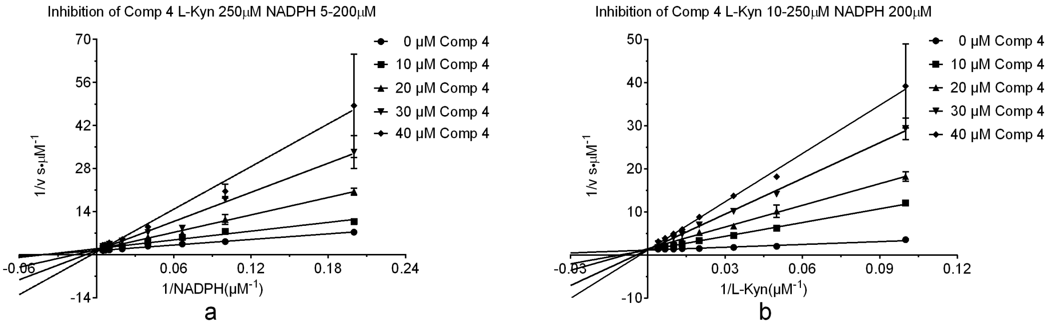

Supplementary Figure 10. Inhibition of *Pf*KMO activity by compound 4 at saturating concentrations of the L-KYN and NADPH substrates. Data are plotted as double reciprocal plots of 1/velocity vs the following: a) 1/[NADPH] at 250 μM L-KYN and increasing concentrations of compound 4; b) 1/[L-KYN] at 200 μM NADPH and increasing concentrations of compound 4.

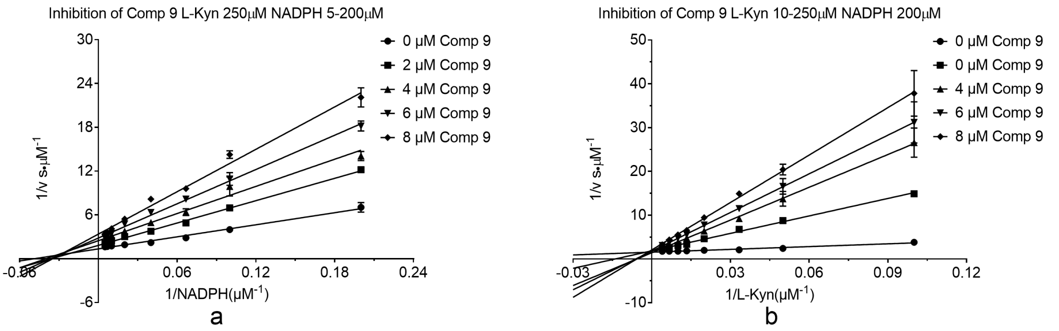

Supplementary Figure 11. Inhibition of *Pf*KMO activity by compound 9 at saturating concentrations of the L-KYN and NADPH substrates. Data are plotted as double reciprocal plots of 1/velocity vs the following: a) 1/[NADPH] at 250 µM L-KYN and increasing concentrations of compound 9; b) 1/[L-KYN] at 200 µM NADPH and increasing concentrations of compound 9.

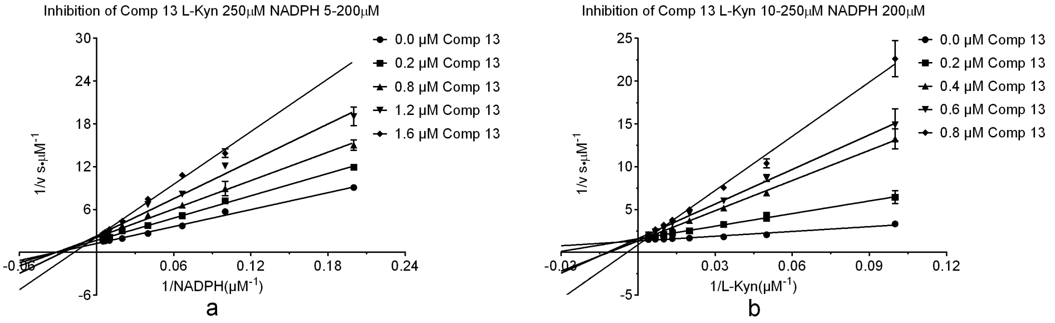

Supplementary Figure 12. Inhibition of *Pf*KMO activity by compound 13 at saturating concentrations of the L-KYN and NADPH substrates. Data are plotted as double reciprocal plots of 1/velocity vs the following: a) 1/[NADPH] at 250 µM L-KYN and increasing concentrations of compound 13; b) 1/[L-KYN] at 200 µM NADPH and increasing concentrations of compound 13.

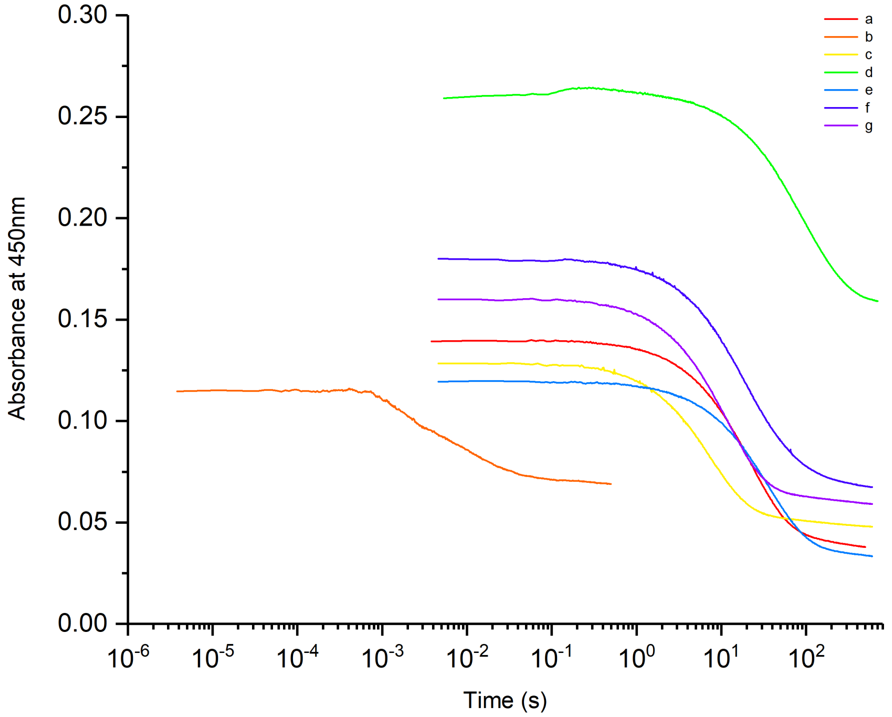

Supplementary Figure 13. Reduction of flavin cofactor by NADPH in the presence and absence of ligands. Anaerobic mixtures of 10 µM *Pf*KMO with or without ligand were mixed against anaerobic 2 mM NADPH on a stopped-flow spectrophotometer at 25℃. **a,** 10 µM *Pf*KMO mixed with 2 mM NADPH; **b**, 10 µM *Pf*KMO mixed with 500 µM L-KYN; **c**, 10 µM *Pf*KMO mixed with 120 µM **1**; **d**, 10 µM *Pf*KMO mixed with 700 µM **4**; **e**, 10µM *Pf*KMO mixed with 60 µM **9**; **f**, 10µM *Pf*KMO mixed with 6 µM **13**; **g**, 10µM *Pf*KMO mixed with 220 µM **1h**.

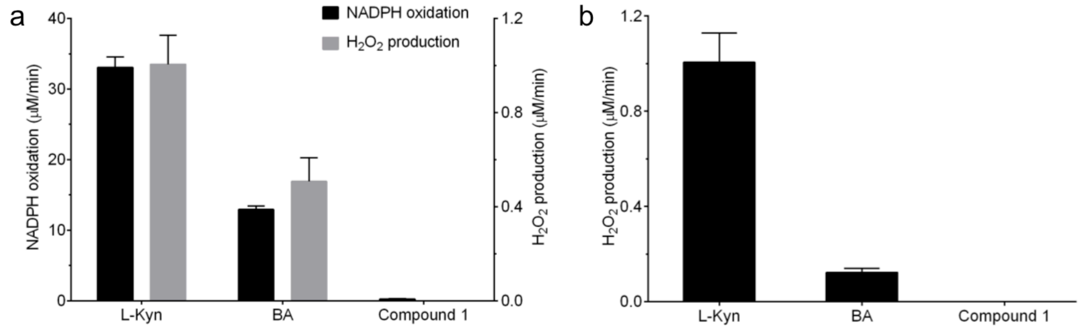

Supplementary Figure 14. Hydrogen peroxide production in PfKMO catalysed reactions. a, NADPH oxidation and H_2O_2 production rates of PfKMO in the presence and absence of KMO inhibitor. The H_2O_2 production rate was measured always in the presence of L-KYN. **b**, The H_2O_2 production rate of BA and compound **1** measured without L-KYN.

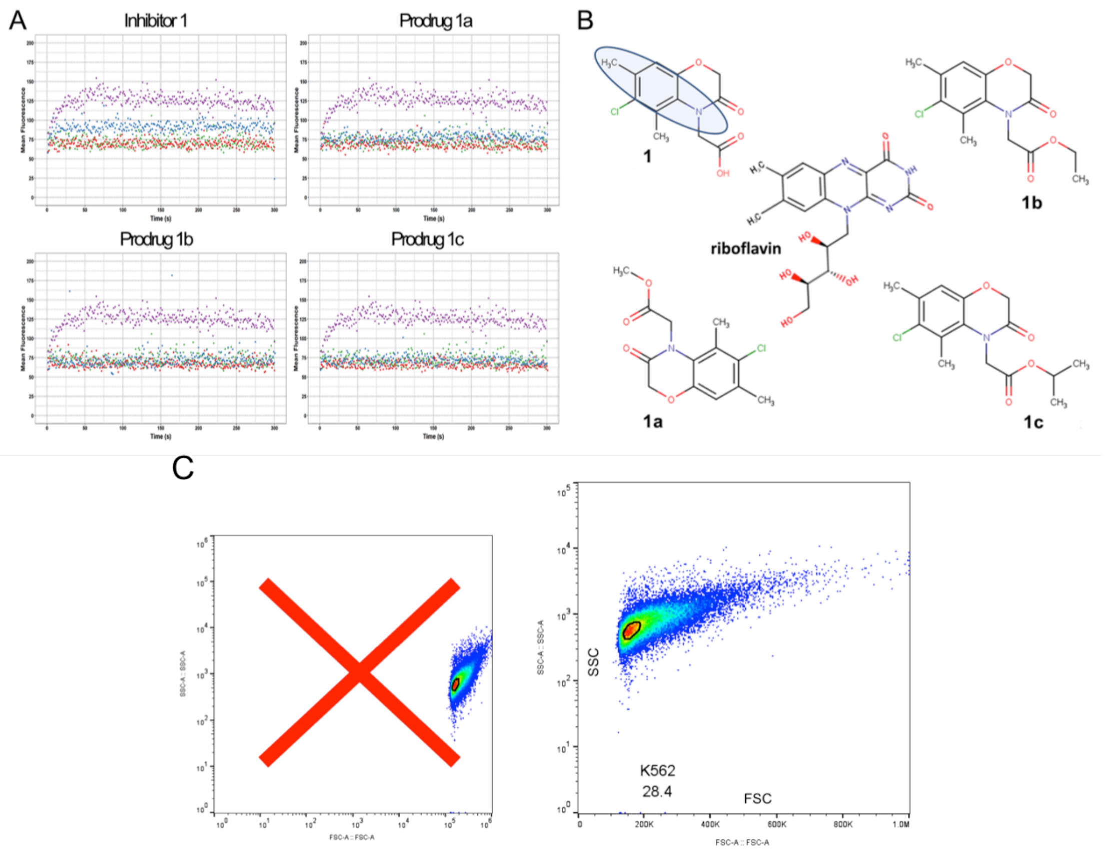

Supplementary Figure 15. A. Uptake of riboflavin into K562 cells. K562 cells were suspended in Dulbecco's phosphate-buffered saline (DPBS) supplemented with 5 mM glucose + 1 % fetal bovine serum (FBS) at a density of 0.5 x 10^6 cells/mL. Compounds were added to Eppendorf tubes and DMSO removed through drying (vacuum centrifuge). Riboflavin was present at 1 µM and inhibitor **1** or prodrugs at 100 µM. 500 µL cells were added to Eppendorf tubes (containing the compound(s) of interest) and fluorescence measured immediately (excitation 488 nm, emission 525 ± 25nm) in a SONY SH800 flow cytometer over 300 s. Cells were maintained at 37 ℃ throughout in a water bath. Each symbol is the mean fluorescence of ~30 cells observed over a period of 1s. Purple, 1 µM riboflavin only; Red, 100 µM compound only; Blue, 1 µM riboflavin plus 100 µM compound; Green, no additions (control). Note that the external concentration of riboflavin was rather critical, as the riboflavin transporters also catalyse exchange reactions with their various substrates. **B.** Structures of KMO inhibitors and prodrugs and riboflavin, and their maximum common substructures (shown as a blue oval on **1**). C. Gating strategy employed in this assay. Gate applied to K562 cell population is marked with a black line. SSC, side scatter, FSC, Forward scatter.

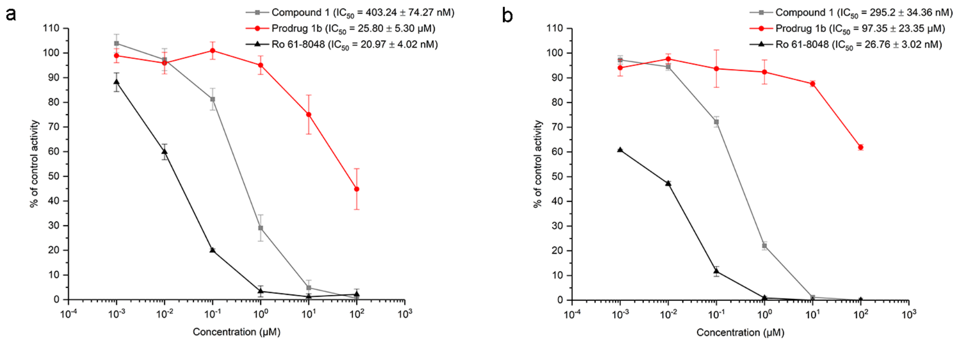

Supplementary Figure 16. Inhibition of KMO activity in rat brain and liver homogenates. KMO activity was measured in rat brain (a) and liver (b) with homogenates treated across a range of concentrations for compound 1, prodrug 1b and Ro 60-8048, respectively. All the data plotted are averages with SEM.

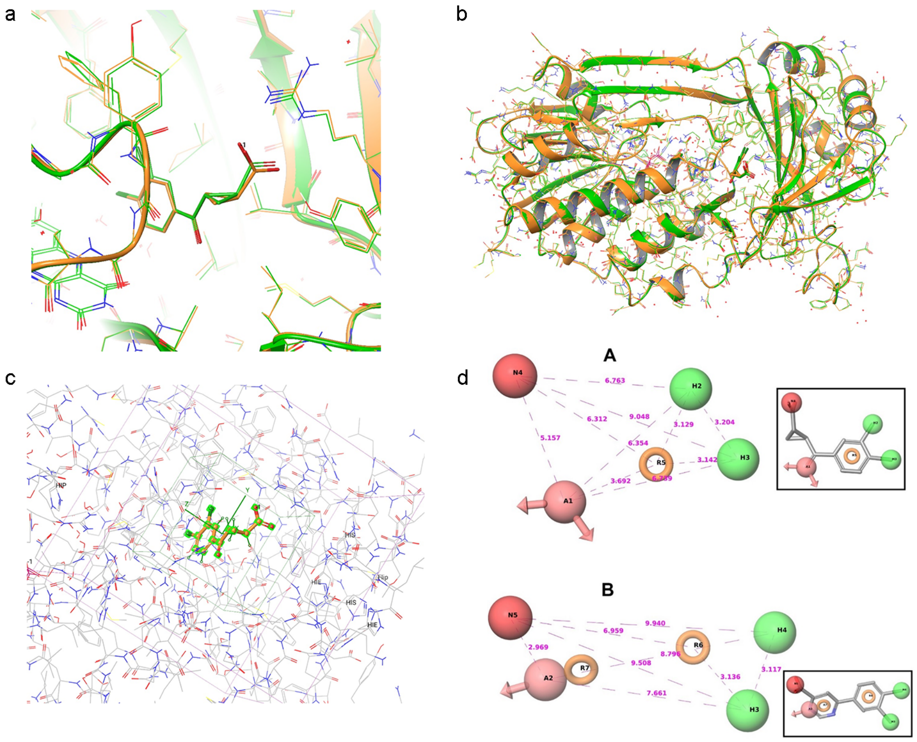

Supplementary Figure 17. Virtual screening to identify new KMO inhibitors. a) Active site superimposition of protein (pre-process in green and post-process in orange). b) Superimposition of the full protein before (green) and after (orange) refinement. c) The grid used for virtual screening (outer purple box). A centroid area in which the diameter midpoint of each docked compound was positioned (inner green box, 10×10×10 Å). d) Ligand-based pharmacophore models. (A) UPF648-based; and (B) Pyrimidine derivative-based. A-acceptor; H-hydrophobic; N-negative charge; and R-aromatic ring. The respective distances between 3D coordinates of features are shown in Å

2. Supplementary Tables

Supplementary Table 1. Rates for the reduction of flavin cofactor by 2 mM NADPH with saturated L-KYN or KMO inhibitors.

2mM NADPH with Complex	Reduction rate (s^{-1})
*Pf*KMO	0.07± 0.001
*Pf*KMO and L-KYN	339± 16.6
*Pf*KMO and **1**	0.13± 0.005
*Pf*KMO and **4**	0.03± 0.005
*Pf*KMO and **9**	0.03± 0.001
*Pf*KMO and **13**	0.06± 0.002
*Pf*KMO and **1h**	0.09± 0.001

Supplementary Table 2. Solubility values for test and control compounds in PBS, pH 7.4 (2% DMSO)

Compound	Solubility(µM)		
	n=1	n=2	Mean
Hydrocortisone	192	187	190
Reserpine	9	9	9
Inhibitor **1**	191	194	193
Inhibitor **3**	68	65	67
Inhibitor **5**	186	183	185
Inhibitor **6**	186	188	187
Inhibitor **8**	193	193	193

Supplementary Table 3. Stability of test and control compounds in mouse hepatic microsomes: half-life and intrinsic clearance values.

Compound	Half-life (minutes)			Mean intrinsic clearance (µL/min/mg microsome protein)
	n=1	n=2	Mean	
Midazolam	<5	<5	<5	>554
Dextromethorphan	28	29	29	97
Inhibitor **1**	>100	>100	>100	<28
Inhibitor **3**	48	69	59	49
Inhibitor **5**	>100	>100	>100	<28
Inhibitor **6**	>100	>100	>100	<28
Inhibitor **8**	>100	>100	>100	<28

Supplementary Table 4. Stability of compounds and positive control in rat hepatocytes.

Compound	Mean intrinsic clearance (mL/min/g liver)
Verapamil	9.9
Compound **1b**	>50
Compound **1**	0.8

Supplementary Table 5. Permeability, recovery and efflux ratios for test and control compounds in wild type MDCK cell lines.

Compound	P_{app} A>B x 10^{-6} cm/sec			P_{app} B>A x 10^{-6} cm/sec			Recovery A>B (%)			Recovery B>A (%)			Mean efflux ratio
	n=1	n=2	Mean	n=1	n=2	Mean	n=1	n=2	Mean	n=1	n=2	Mean	
Propranolol	52	57	54.5	30	31	30.5	88	94	91	77	84	80.5	0.56
Vinblastine	<1	<1	<1	3.6	3.9	3.75	48	45	46.5	71	74	72.5	>3.8
Inhibitor **1**	16	11	13.5	2.6	2.3	2.45	100	87	93.5	108	109	108.5	0.18
Inhibitor **3**	2.7	3.9	3.3	2.7	3.2	2.95	15	15	15	50	48	49	0.89
Inhibitor **5**	2.6	2.1	2.35	1.6	1.4	1.5	81	81	81	86	81	83.5	0.62
Inhibitor **6**	33	41	37	51	50	50.5	57	75	66	98	89	93.5	1.36
Inhibitor **8**	20	15	17.5	6.5	3.6	5.05	102	92	97	90	95	92.5	0.28

Supplementary Table 6. Permeability, recovery and efflux ratios for test and control compounds in MDR1 MDCK cell lines

Compound	P_{app} A>B x 10^{-6} cm/sec			P_{app} B>A x 10^{-6} cm/sec			Recovery A>B (%)			Recovery B>A (%)			Mean efflux ratio
	n=1	n=2	Mean	n=1	n=2	Mean	n=1	n=2	Mean	n=1	n=2	Mean	
Propranolol	53	52	52.5	39	37	38	97	88	92.5	83	78	80.5	0.72
Vinblastine	<1	<1	<1	24	20	22	55	55	55	71	64	67.5	>22
Inhibitor **1**	18	14	16	1.8	2.4	2.1	107	91	99	62	106	84	0.14
Inhibitor **3**	2.9	3.6	3.25	4.9	5	4.95	15	21	18	51	48	49.5	1.5
Inhibitor **5**	2.8	2.4	2.6	2.4	2.1	2.25	87	79	83	80	83	81.5	0.88
Inhibitor **6**	40	61	50.5	51	32	41.5	68	96	82	93	67	80	0.81
Inhibitor **8**	19	19	19	4	5.9	4.9	97	97	97	70	83	76.5	0.26

Supplementary Table 7. Brain penetration of inhibitor **1** and its derivatives

Compound	Mouse	Time (min)	In blood (ng/mL)	In brain (ng/g)	Brain:Blood Ratio	Mean Brain:Blood ratio
Inhibitor **1**	1	5	3059	100	0.03	0.03
	2		4036	116	0.03	
	3		4260	121	0.03	
	4	60	34	≤ LLoQ	ND	NR
	5		44	≤ LLoQ	ND	NR
	6		86	≤ LLoQ	ND	NR
Inhibitor **1** from prodrug **1a**	1	5	906	1159	1.28	1.42(n=2)
	2		238	467	2	
	3		≤ LLoQ	28	ND	
	4	60	226	107	0.47	0.47
	5		185	78	0.42	
	6		167	85	0.51	
Inhibitor **1** from prodrug **1b**	1	5	811	1570	1.94	1.95
	2		614	960	1.56	
	3		636	1495	2.35	
	4	60	72	53	0.74	0.38
	5		231	45	0.19	
	6		236	48	0.2	
Inhibitor **1** from prodrug **1c**	1	5	131	42	0.32	0.38
	2		108	49	0.45	
	3		75	29	0.38	
	4	60	14	≤ LLoQ	ND	NR
	5		21	≤ LLoQ	ND	NR
	6		37	≤ LLoQ	ND	NR
Inhibitor **1** from prodrug **1d**	1	5	≤ LLoQ	≤ LLoQ	ND	NR
	2		≤ LLoQ	≤ LLoQ	ND	NR
	3		≤ LLoQ	≤ LLoQ	ND	NR
	4	60	≤ LLoQ	≤ LLoQ	ND	NR
	5		≤ LLoQ	≤ LLoQ	ND	NR
	6		16	≤ LLoQ	ND	NR
Inhibitor **1h**	1	5	1626	30	0.02	0.01
	2		1891	15	0.01	
	3		1965	26	0.01	
	4	60	≤ LLoQ	≤ LLoQ	ND	ND
	5		≤ LLoQ	≤ LLoQ	ND	ND
	6		≤ LLoQ	≤ LLoQ	ND	ND

LLoQ: Lower Limit of Quantification. LLoQ (blood) = 1 ng/mL, LLoQ (brain) = 4 ng/g, NR = no result, ND = not determined due to analytical issues.

Supplementary Table 8. Elimination of inhibitor **1** in urine following single intravenous administration at 1 mg free base/kg to a female C57BL/6 mouse.

	Time	Absolute amount of drug recovered in urine (μg)	Amount of drug in urine recovered (% Dose)
Inhibitor **1**	0 - 8 h	8.67	30.3
	8 - 24 h	0.15	0.5

Supplementary Table 9. Brain penetration of prodrug **1b** in the rat

Rat	Time (min)	In blood (ng/mL)	Unbound blood (ng/mL)	In brain (ng/g)	Unbound brain (ng/g)	Brain:Blood Ratio	Mean Brain:Blood ratio
1		≤ 3		2346	38	ND	
2	5	≤ 3	N/A	2623	42	ND	NR
3		≤ 3		2264	36	ND	
4		≤ 3		829	13	ND	
5	15	≤ 3	N/A	715	11	ND	NR
6		≤ 3		791	13	ND	
7		≤ 3		240	4	ND	
8	30	≤ 3	N/A	ND	ND	ND	NR
9		≤ 3		203	3	ND	
10		≤ 3		ND	ND	ND	
11	60	≤ 3	N/A	ND	ND	ND	NR
12		≤ 3		62	1	ND	

Blood: LLoQ = 1 ng/mL; Scaled LLoQ = dilution factor * LLoQ = 3*1 = 3 ng/mL; Higher Limit of Quantification (HLoQ) = 1000ng/mL. **Brain:** LLoQ = 20 ng/mL; LLoQ brain scaled (ng/g) = LLoQ * 3 = 20*3= 60 ng/g; HLoQ= 1000 ng/mL. Fraction unbound (blood) = n/a; Fraction unbound (brain) = 0.016.

Supplementary Table 10. Brain and blood levels of inhibitor **1** released from prodrug **1b** in the rat.

Rat	Time (min)	In blood (ng/mL)	Unbound blood (ng/mL)	In brain (ng/g)	Unbound brain (ng/g)	Brain:Blood Ratio	Mean Brain:Blood ratio
1		5054	202	7403	2221	1.46	
2	5	6942	278	6757	2027	0.97	1.28
3		3380	135	4741	1422	1.4	
4		3035	121	9699	2910	3.2	
5	15	2128	85	6790	2037	3.19	3.22
6		2786	111	9087	2726	3.26	
7		2924	117	4057	1217	1.39	
8	30	2687	107	6044	1813	2.25	1.78
9		1996	80	3399	1020	1.7	
10		1289	52	749	225	0.58	
11	60	1080	43	ND	ND	NR	0.37
12		1269	51	662	199	0.52	

Blood: LLoQ = 1ng/mL; Scaled LLoQ = dilution factor * LLoQ = 3*1= 3 ng/mL; HLoQ = 5000 ng/mL. **Brain:** LLoQ = 200 ng/mL ± 20%, LLoQ brain scaled (ng/g) = LLoQ * 3 = 200*3 = 600 ng/g, HLoQ = 5000ng/mL. Fraction unbound (blood) = 0.040. Fraction unbound (brain) = 0.30.

Supplementary Table 11. List of target inhibitor compounds together with chemical and supplier.

Compound	Chemical name	Supplier
**1**	(6-chloro-5,7-dimethyl-3-oxo-2,3-dihydro-4*H*-1,4-benzoxazin-4-yl) acetic acid	Chembridge
**2**	3-[1-(4-bromophenyl)-5-(2-thienyl)-1*H*-pyrrol-2-yl] propanoic acid	Chembridge
**3**	4-(2-phenyl[1,2,4]triazolo[5,1-b][1,3]thiazol-6-yl)benzene-1,3-diol	Maybridge
**4**	(*R*)-2-(5-chloro-1-methyl-1*H*-indole-2-carboxamido)-2-phenylacetic acid	Interbioscreen
**5**	(*R*)-2-(5-fluoro-1-methyl-1*H*-indole-2-carboxamido)-2-phenylacetic acid	Interbioscreen
**6**	3-[(4-chlorophenyl)thio]-2-methylpropanoic acid	Maybridge
**7**	(*R*)-2-(1,5-dimethyl-1*H*-indole-2-carboxamido)-2-phenylacetic acid	Interbioscreen
**8**	3-(6-chloro-3-oxo-2,3-dihydro-4*H*-1,4-benzoxazin-4-yl) propanoic acid	Vitas-M
**9**	5-(2,3-dichlorophenyl) furan-2-carboxylic acid	InterBioScreen
**10**	2-(5-(2,4-dichlorophenyl)furan-2-carboxamido)benzoic acid	InterBioScreen
**11**	5-[2-(trifluoromethyl)phenyl]furan-2-carboxylic acid	Vitas-M
**12**	5-[2-chloro-5-(trifluoromethyl)phenyl]furan-2-carboxylic acid	Vitas-M
**13**	5-(3,4-dichlorophenyl) furan-2-carboxylic acid	Vitas-M
**14**	3-[5-(4-chlorophenyl)-1-(methylethyl)pyrrol-2-yl]propanoic acid	TimTec
**15**	2-(5-(4-methoxyphenyl)-2-oxopyrazin-1(2*H*)-yl)acetic acid	InterBioScreen
**16**	5-(2,4-dichlorophenyl) furan-2-carboxylic acid	Vitas-M
**17**	5- (3-chlorophenyl) furan-2-carboxylic acid	Vitas-M
**18**	5-(3-chloro-4-fluorophenyl) furan-2-carboxylic acid	Vitas-M
**19**	5-(4-bromo-3-chlorophenyl) furan-2-carboxylic acid	Vitas-M

Supplementary Table 12. Data collection and refinement statistics for *Pf*KMO crystal structures.

	Inhibitor 1*	Inhibitor 9	Inhibitor 4	Inhibitor 13	Inhibitor 1h	KYN
**Data collection**						
Space group	P 1 21 1	P 1 21 1	P 1 21 1	P 1 21 1	P 1 21 1	P 1 21 1
Cell dimensions						
a, *b*, *c* (Å)	71.08, 52.92, 137.46	69.91, 53.03, 136.03	74.592, 46.577, 133.996	69.54, 52.19, 136.21	70.351, 53.253, 135.844	69.908, 52.542, 136.173
α, β, γ (°)	90, 104.08, 90	90, 103.81, 90	90, 104.76, 90	90, 103.53, 90	90, 103.812, 90	90, 104.142, 90
Resolution (Å)	19.98 - 1.97 (2.04 - 1.97)**	132.1 - 1.63 (1.688 - 1.63)	72.13 - 2.029 (2.102 - 2.029)	66.21 - 2.15 (2.227 - 2.15)	28.75 - 2.0 (2.071 - 2.0)	42.4 - 1.947 (2.017 - 1.947)
R_{sym} or R_{merge}	0.08 (0.83)	0.05683 (0.6193)	0.06373 (0.5658)	0.06293 (0.601)	0.05622 (0.5078)	0.0617 (0.1846)
$I / \sigma I$	11.07 (1.54)	10.99 (1.49)	12.48 (2.30)	12.94 (2.02)	13.72 (2.30)	14.28 (5.43)
Completeness (%)	98.20 (99.08)	96.96 (86.92)	98.29 (97.95)	99.19 (99.81)	99.72 (99.92)	98.07 (97.60)
Redundancy	3.4 (3.4)	3.0 (2.1)	3.4 (3.5)	3.2 (3.4)	3.4 (3.5)	4.1 (4.0)
**Refinement**						
Resolution (Å)	19.98 - 1.97 (2.04 - 1.97)	132.1 - 1.63 (1.688 - 1.63)	72.13 - 2.029 (2.102 - 2.029)	66.21 - 2.15 (2.227 - 2.15)	28.75 - 2.0 (2.071 - 2.0)	42.4 - 1.947 (2.017 - 1.947)
No. reflections	69316 (6912)	117484 (10464)	57247 (5601)	51796 (5188)	66340 (6576)	69311 (6802)
R_{work} / R_{free}	0.1797/0.2145	0.1801/0.2095	0.1681/0.2175	0.1915/0.2337	0.1787/0.2148	0.1664/0.2161
No. atoms						
Protein	7020	6865	6925	6855	6942	7069
Ligand/ion	179	139	161	138	148	106
Water	435	395	346	186	477	808
B-factors						
Protein	34.58	27.33	41.91	52.15	40.12	26.28
Ligand/ion	26.27	17.32	42.68	33.29	31.05	19.55
Water	42.41	29.91	44.53	45.85	40.64	34.49
R.m.s. deviations						
Bond lengths (Å)	0.014	0.013	0.007	0.012	0.002	0.017
Bond angles (°)	1.16	1.19	0.86	1.07	0.56	1.42

Supplementary Table 13. K_i values of KMO inhibitors.

KMO inhibitor	K_i (μM)
**1**	0.50 ± 0.06
**4**	3.00 ± 0.24
**9**	0.15 ± 0.01
**13**	0.02 ± 0.001
**1h**	1.06 ± 0.04

3. Supplementary Methods

3.1 Virtual screening to identify new KMO inhibitors.

Potential KMO inhibitors were identified by BioFocus (Charles Rivers Laboratories). This was performed by structure-based virtual screening (Scheme 1) against the 4J36[2] (Protein Data Bank (PDB) structure) using Glide (Schrodinger). In addition, a ligand-based virtual screening (Scheme 2) was carried out against the bound conformation of UPF648 inhibitor using ROCS (OpenEye). This led to the identification of more than 1,000 compounds, which were selected for screening against KMO activity.

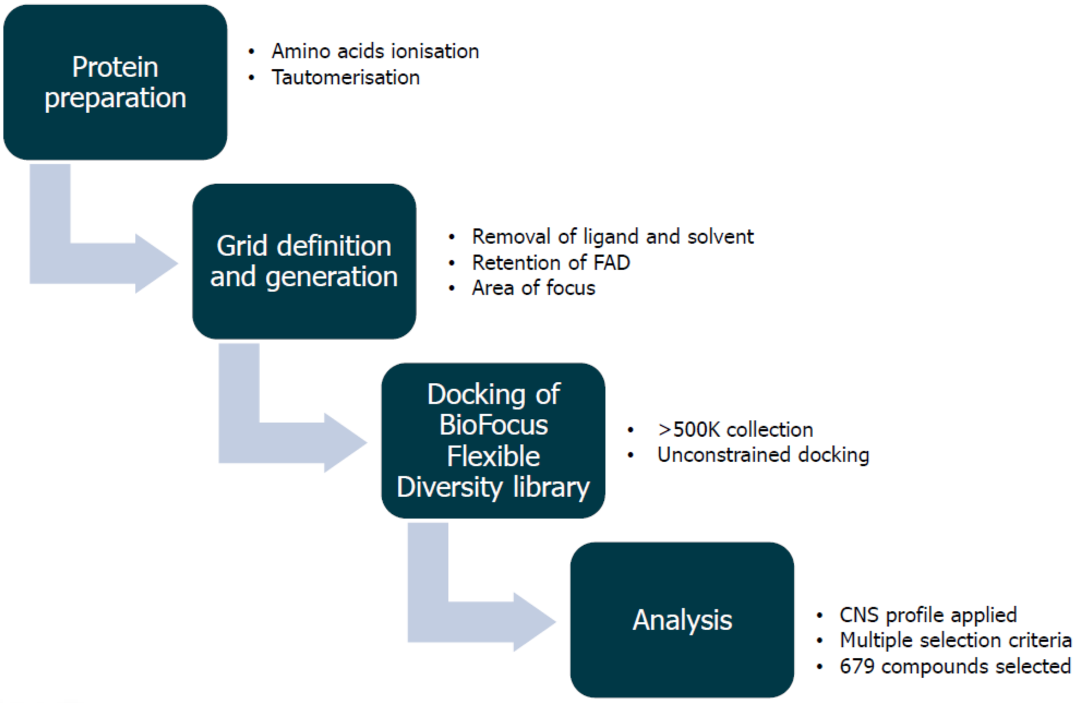

Scheme 1. Overview of the structure-based screening approach (BioFocus, Charles Rivers Laboratories) used to identify potential KMO inhibitors.

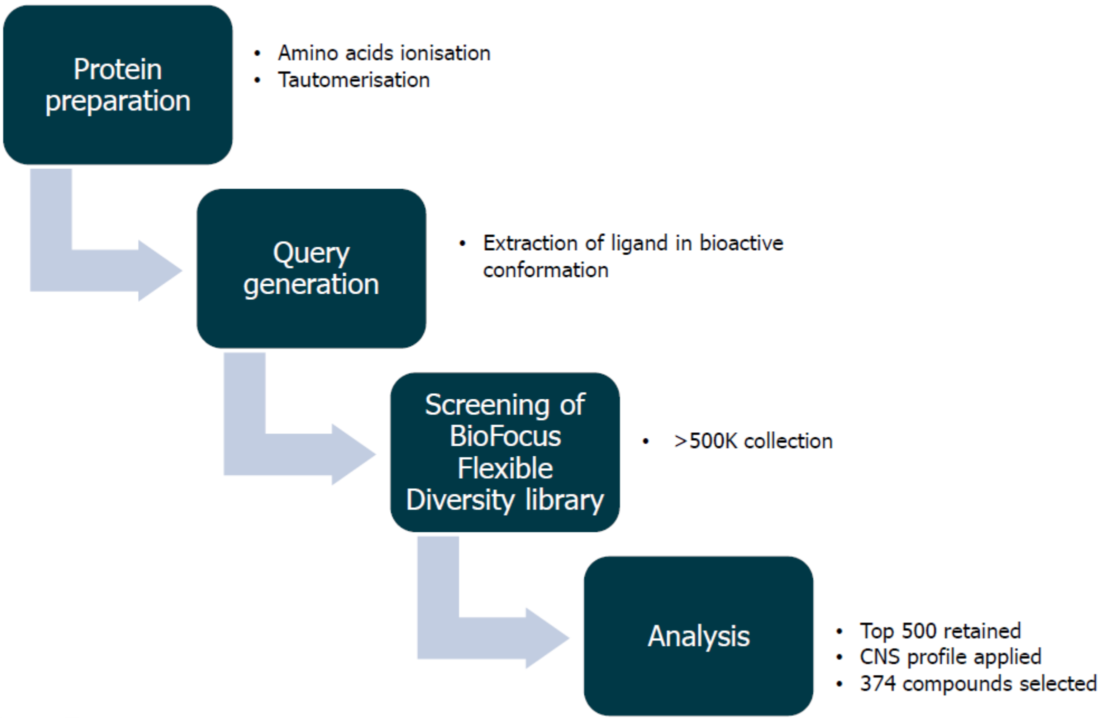

Scheme 2. Overview of the ligand-based screening approach (BioFocus, Charles Rivers Laboratories) used to identify potential KMO inhibitors.

In a separate approach a total of 592,701 structures from InterBioScreen[3], ChemBridge[4], Maybridge[5], and MyriaScreen[6] databases, were subjected to the Phase database preparation module (Schrödinger)[7]. Virtual screening (Scheme 3) was performed using Maestro (version 9.9), implemented from Schrödinger molecular modelling suite-2014-3[7]. The raw protein structure of KMO co-crystallised with tightly bound inhibitor UPF648 (PDB entry 4J36) was prepared by giving preliminary treatment including adding hydrogens, refining the loop, and finally minimising using an OPLS-2005 force field, The RMSD between the Cα of the raw protein structure and the refined protein structure was 0.1602 Å, indicating only very minor structural changes (Supplementary Figure 17a and 17b). A grid with dimensions of 20×20×20 Å was generated for docking analysis, using the coordinates of UPF648 as reference points (Supplementary Figure 17c). The grid was much bigger than UPF648, which is 9 Å in diameter, and large enough to accommodate database compounds with desired features. The following three step docking protocol was implemented using Glide (Schrödinger): (1) high-throughput virtual screening (HTVS) of 592,701 structures; (2) standard precision (SP) docking of the top 10% of structures based on docking score from HTVS; and (3) extra-precision docking of the top 5% of

structures based on docking score from SP docking. This resulted in the selection of 268 structures with GScore < -9.0 for further analysis. A complementary approach involved ligand-based virtual screening in tandem with structure-based virtual screening. The bound conformation of UPF648[8] (PDB entry 4J36) and the energy minimized conformation (LigPrep, Schrödinger) of a previously reported pyrimidine analogue (6-(3,4-dichlorophenyl) pyrimidine-4-carboxylic acid)[9] were used to develop 3D ligand-based pharmacophores with Phase.[10] The pharmacophore features of these molecules were defined as hydrogen bond acceptor (A), hydrophobic (H), negative charge (N), and aromatic ring (R), which produced five-point (AHHNR) and six-point (AHHNRR) pharmacophores derived from UPF648 and the pyrimidine analogue, respectively (Supplementary Figure 16d). Ligand-based virtual screening of the 592,701 structures was conducted, and structures matching a minimum of three pharmacophore features were kept for calculation of fitness scores. Based on the fitness score, the top 5,000 hits proceeded to structure-based virtual screening by standard precision (SP) docking, followed by extra-precision docking of the top 1,724 structures according to docking score from SP docking. This resulted in the selection of the top 200 structures for further analysis. The final selection of 41 hits for enzymatic studies, from the 468 shortlisted structures, was based on visualisation of binding modes with the key catalytic residues Arg83 and Tyr97, and drug-likeness (Lipinski's rule).

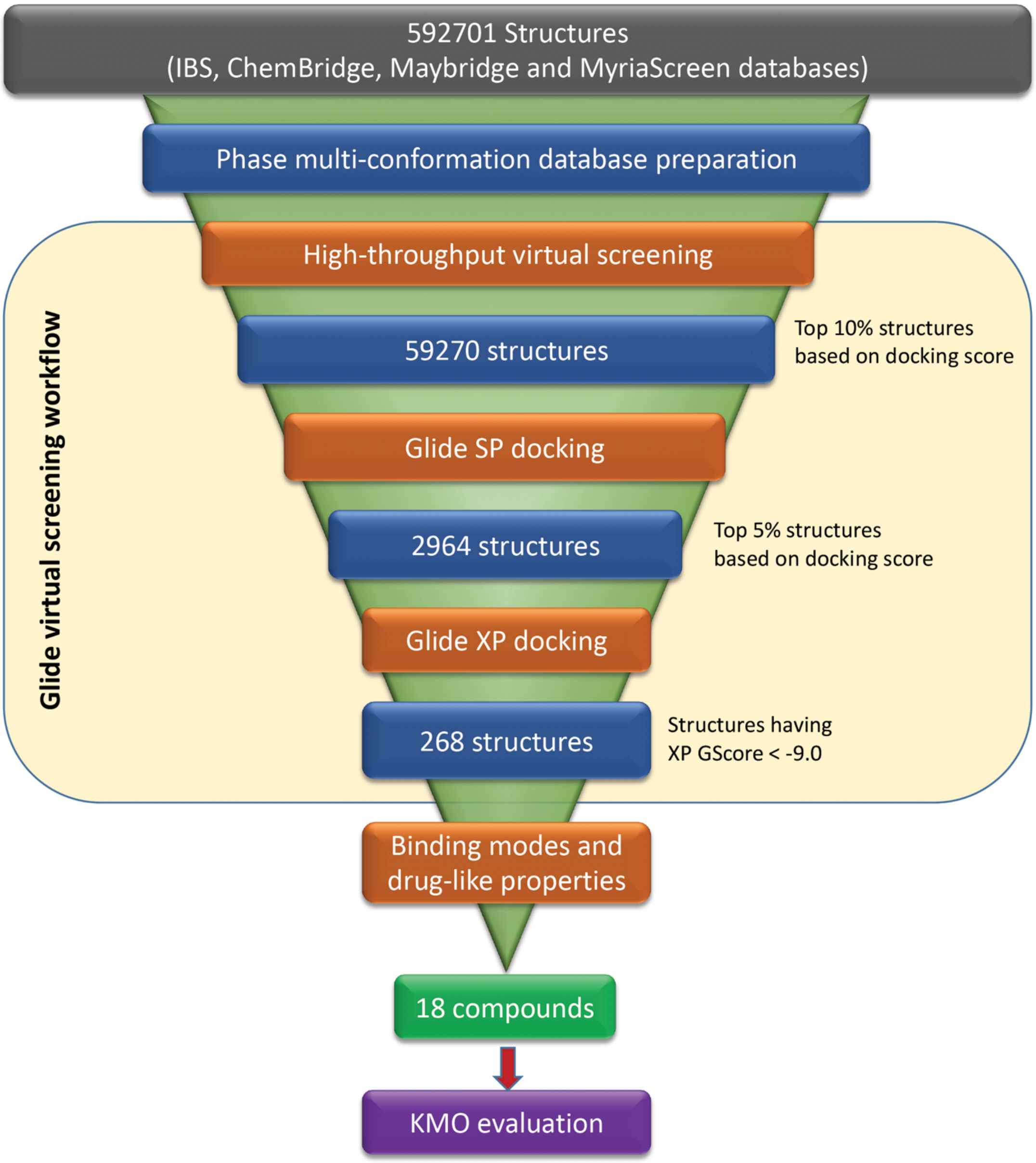

Scheme 3. Overview of the high-throughput virtual screening approach used to identify potential KMO inhibitors.

3.2 General experimental details for synthesis of inhibitor 1 derivatives

All reactions were performed in flame-dried glassware fitted with rubber septa under a positive pressure of nitrogen, unless otherwise noted. Air- and moisture-sensitive liquids were transferred via syringe or stainless steel cannula through rubber septa. Solids were added under inert gas counter flow or were dissolved in appropriate solvents. Low temperature-reactions were carried out in a Dewar vessel filled with a cooling agent: acetone/dry ice (−78 ˚C), H_2O/ice (0 ˚C). Reaction temperatures above room temperature were conducted in a heated oil bath. The reactions were

magnetically stirred and monitored by liquid chromatography mass spectrometry (LCMS) or analytical thin-layer chromatography (TLC), using aluminium plates pre-coated with silica gel (0.25 mm, 60-Å pore size) impregnated with a fluorescent indicator (254 nm). TLC plates were visualized by exposure to ultraviolet light (UV), were stained by submersion in aqueous potassium permanganate solution ($KMnO_4$), ceric ammonium molybdate solution (CAM) or p-anisaldehyde solution (Anis), and were developed by heating with a heat gun. Flash-column chromatography on silica gel was performed using silica gel (60 Å, 40–63 μm). The yields refer to pure material analysed by chromatography and nuclear magnetic resonance spectroscopy (^1H and ^{13}C). Synthesis was performed as a contract service by WuXi AppTec (Shanhai, China).

3.2.1 Materials

Tetrahydrofuran (THF) and diethyl ether (Et_2O) were distilled under N_2 atmosphere from sodium and benzophenone prior to use. Dichloromethane (CH_2Cl_2), triethylamine (Et_3N), diisopropylamine (DIPA) and Hünig's base (DIPEA) were distilled under nitrogen atmosphere from CaH_2 prior to use. Dimethyl sulfoxide (DMSO), acetonitrile (ACN), acetone, toluene, chloroform ($CHCl_3$) and methanol (MeOH) were purchased from Acros Organics as 'extra dry' reagents and used as received. All other reagents and solvents were purchased from chemical suppliers (Sigma-Aldrich, Acros Organics, Alfa Aesar, Strem Chemicals, ABCR) and were used as received. Solvents for extraction, crystallization and flash-column chromatography on silica gel were purchased in technical grade and distilled under reduced pressure prior to use.

3.2.2 Nuclear magnetic resonance (NMR) spectroscopy

NMR spectra were measured on a Bruker 400 MHz spectrometer. Proton chemical shifts are expressed in parts per million (ppm, δ scale) and are referenced to residual proton in the NMR solvent ($CHCl_3$: δ 7.26, CD_3OD: δ 3.34). ^1H NMR spectroscopic data are reported as follows: Chemical shift in ppm (multiplicity, coupling constants *J* (Hz), integration intensity, assigned proton). The multiplicities are abbreviated with s (singlet), br s (broad singlet), d (doublet), t (triplet), q (quartet) and m (multiplet). In case of combined multiplicities, the multiplicity with the larger coupling constant is stated first. Except for multiplets, the chemical shift of all signals, as well for

centrosymmetric multiplets, is reported as the centre of the resonance range. Additional to ^{1}H measurements, 2D NMR techniques such as homonuclear correlation spectroscopy (COSY), total correlation spectroscopy (TOCSY), heteronuclear single quantum coherence (HSQC) and heteronuclear multiple bond coherence (HMBC) were used to assist signal assignment. Coupling constants *J* are reported in Hz.

3.2.3 Liquid Chromatography Mass Spectrometry (LCMS)

LCMS spectra were recorded on an Agilent Technologies 1200 + 6110MS. The following methods were used.

Method name:	**5-95AB**						
Instrument:	**Agilent 1200 & G6110A**						
Column:	**Chromolith Flash RP-18, 25-2 mm**						
Column temperature:	**40 °C**						
Mobile phase A(MPA)	**H_2O + 0.037% (v/v) TFA**						
Mobile phase B(MPB)	**ACN + 0.018% (v/v) TFA**						
Flow rate:	**1.5 mL/min**						
Gradient Ratio:	**Time (min)**	**0.01**	**0.70**	**1.15**	**1.16**	**1.60**	
	**MPA (%)**	**95**	**5**	**5**	**95**	**95**	
	**MPB (%)**	**5**	**95**	**95**	**5**	**5**	
Detection:	**220 nm 254 nm**						
MS Mode:	**Positive**						
MS Range:	**100-1000**						

Method name:	**10-80CD**						
Instrument:	**Agilent 1200 & G6110A**						
Column:	**Xbridge Shield RP18 2.1×50 mm, 5 µm**						
Column temperature:	**40 °C**						
Mobile phase A(MPA)	**H_2O + 10 mM NH_4HCO_3**						
Mobile phase B(MPB)	**ACN**						
Flow rate:	**1.0 mL/min**						
Gradient Ratio:	**Time (min)**	**0.00**	**0.70**	**1.10**	**1.11**	**2.00**	**2.20**
	**MPA (%)**	**90**	**20**	**20**	**90**	**90**	**90**
	**MPB (%)**	**10**	**80**	**80**	**10**	**10**	**10**

Detection:	**220 nm**
MS Mode:	**Positive**
MS Range:	**100-1000**

Method name:	**WUXIAB10**						
Instrument:	**Agilent 1200 & 1956A**						
Column:	**Luna-C18 (2) 2.0×50 mm, 5µm**						
Column temperature:	**40 ˚C**						
Mobile phase A(MPA)	**H_2O + 0.037% (v/v) TFA**						
Mobile phase B(MPB)	**ACN + 0.018% (v/v) TFA**						
Flow rate:	**0.8 mL/min**						
Gradient Ratio:	**Time (min)**	**0.00**	**0.40**	**3.40**	**3.85**	**3.86**	**4.50**
	**MPA (%)**	**90**	**90**	**0**	**0**	**90**	**90**
	**MPB (%)**	**10**	**10**	**100**	**100**	**10**	**10**
Detection:	**220 nm**						
MS Mode:	**Positive**						
MS Range:	**100-1000**						

3.2.4 High-performance liquid chromatography (HPLC)

HPLC was recorded on either a Shimadzu LC-20AB or a Shimadzu LC-20AD. The following methods were used.

Method name:	**10-80HPLC**						
Instrument:	**Shimadzu 20AB**						
Column:	**Luna-C18 (2) 2.0×50 mm, 5 µm**						
Column temperature:	**40 ˚C**						
Mobile phase A(MPA)	**H_2O + 0.037% (v/v) TFA**						
Mobile phase B(MPB)	**ACN + 0.018% (v/v) TFA**						
Flow rate:	**0.8 mL/min (0.01-4.90 min), 1.2 mL/min (4.93-5.50 min)**						
Gradient Ratio:	**Time (min)**	**0.01**	**4.00**	**4.90**	**4.92**	**5.50**	
	**MPA (%)**	**90**	**20**	**20**	**90**	**90**	
	**MPB (%)**	**10**	**80**	**80**	**10**	**10**	
Detection:	**220 nm 215 nm 254 nm**						

Method name:	**10-80HPLC(CD)**						
Instrument:	**Shimadzu 20AB**						
Column:	**XBridge Shield RP18 2.1×50 mm, 5 µm**						
Column temperature:	**40 ˚C**						
Mobile phase A(MPA)	**H_2O + 10 mM NH_4HCO_3**						
Mobile phase B(MPB)	**ACN**						
Flow rate:	**0.8 mL/min (0.01-4.90 min, 1.2 mL/min (4.93-5.50 min)**						
Gradient Ratio:	**Time (min)**	**0.01**	**4.00**	**4.90**	**4.92**	**5.50**	
	**MPA (%)**	**90**	**20**	**20**	**90**	**90**	
	**MPB (%)**	**10**	**80**	**80**	**10**	**10**	
Detection:	**220 nm 215 nm 254 nm**						

3.3 Synthesis of inhibitor 1 derivatives

3.3.1 4-Chloro-3,5-dimethyl-2-nitro-phenol

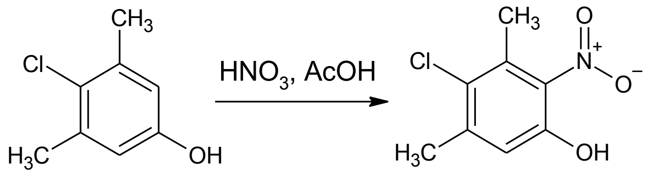

To a solution of 4-chloro-3,5-dimethyl-phenol (30 g, 192 mM, 1 eq) in acetic acid (450 mL) was added HNO_3 (19 g, 192 mM, 13 mL, 65% purity, 1 eq) at 5 ˚C and the mixture stirred at 25 ˚C for 1 h. TLC (25% v/v ethyl acetate in petroleum ether) showed the reaction to be complete. The reaction mixture was poured into ice-water (500 g) and stirred for 10 min. The resulting solid was collected by filtration, washed with water (200 mL × 2) and dried in high vacuum at 50 ˚C for 24 h. 4-Chloro-3,5-dimethyl-2-nitro-phenol (36 g, 179 mM, 93.2% yield) was obtained as an orange solid.

TLC (25% v/v ethyl acetate in petroleum ether): R_f = 0.45 ($KMnO_4$, UV);
^{1}H NMR ($CDCl_3$, 400 MHz): δ = 9.54 (s, 1 H), 6.92 (s, 1 H), 2.61 (s, 3 H), 2.40 (s, 3 H).

3.3.2 Methyl 2-(4-chloro-3,5-dimethyl-2-nitro-phenoxy)acetate

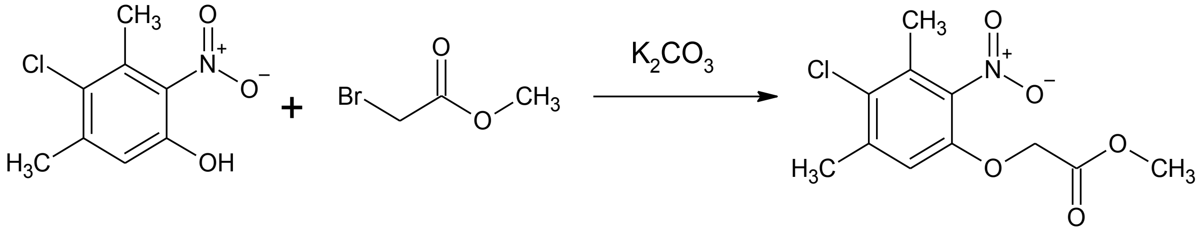

To a solution of 4-chloro-3,5-dimethyl-2-nitro-phenol (36 g, 179 mM, 1 eq) and methyl 2-bromoacetate (33 g, 214 mM, 20 mL, 1.2 eq) in DMF (150 mL) was added K_2CO_3 (37 g, 268 mM, 1.5 eq) at 25 ˚C and the mixture heated to 80 ˚C and stirred for 2 h. TLC (25% v/v ethyl acetate in petroleum ether) showed the reaction to be complete. The reaction mixture was cooled and poured into water (500 mL). The resulting mixture was stirred for 10 minutes and filtered and the resulting solid collected by filtration and washed with water (200 mL x 2). The solid was further dried in high vacuum at 50 ˚C for 24 h. Methyl 2-(4-chloro-3,5-dimethyl-2-nitro-phenoxy)acetate (40 g, 140 mM, 78.5% yield, 96.9% purity) was obtained as a yellow solid.

TLC (25% ethyl acetate in petroleum ether): R_f = 0.40 ($KMnO_4$, UV);

^{1}H NMR ($CDCl_3$, 400 MHz): δ = 6.68 (s, 1 H), 4.68 (s, 2 H), 3.79 (s, 3 H), 2.39 (s, 3 H), 2.32 (s, 3 H);

HPLC (Method 10-80HPLC): R_t = 2.84 min, 96.9% purity.

3.3.3 6-Chloro-5,7-dimethyl-4H-1,4-benzoxazin-3-one

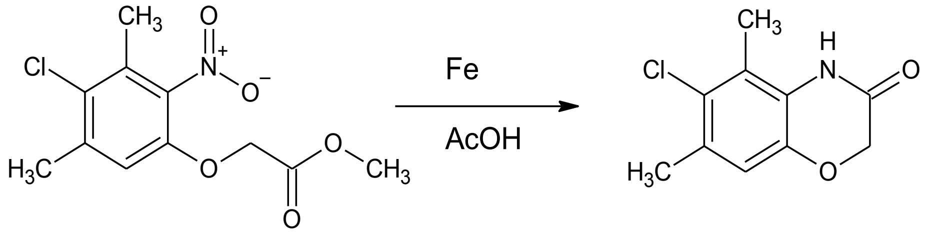

Iron powder (82 g, 1.5 M, 10 eq) was added portion wise to a solution of methyl 2-(4-chloro-3,5-dimethyl-2-nitro-phenoxy)acetate (40 g, 146 mM, 1 eq) in AcOH (500 mL) at 80 ˚C over 30 min. The mixture was heated to 110 ˚C and stirred for an

additional 30 min. TLC and LCMS showed the reaction was complete. The reaction mixture was filtered through a pad of celite and the filter cake washed with hot AcOH (250 mL x 2). The filtrate was concentrated under vacuum to give a residue which was further diluted with water (500 mL) and stirred for 10 min. The resulting precipitate was collected by filtration and washed with water (250 mL x 2) and dried in high vacuum at 50 ˚C for 48 hours and 6-chloro-5,7-dimethyl-4*H*-1,4-benzoxazin-3-one (26 g, 123 mM, 84.1% yield) was obtained as a white solid.

TLC (20% v/v ethyl acetate in petroleum ether): R_f = 0.30 ($KMnO_4$, UV);
LCMS: Rt = 0.74 min, MH^+ = 212.

3.3.4 Methyl 2-(6-chloro-5,7-dimethyl-3-oxo-1,4-benzoxazin-4-yl) acetate (1a)

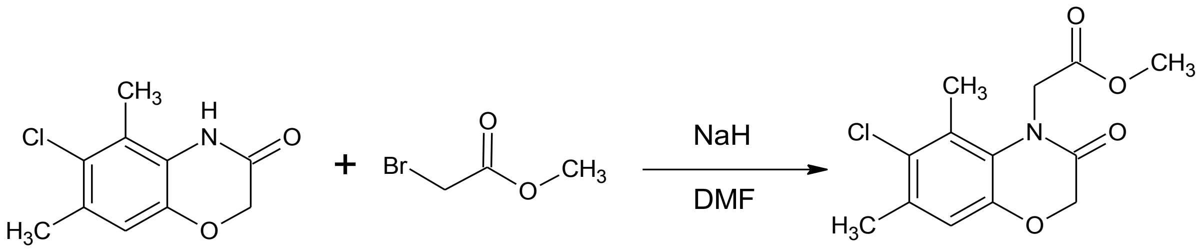

To a solution of 6-chloro-5,7-dimethyl-4H-1,4-benzoxazin-3-one (10 g, 47 mM, 1 eq) in DMF (70 mL) was added NaH (4 g, 95 mM, 60% purity, 2 eq) at 25 ˚C and the resultant mixture stirred at 25 ˚C for 0.5 h. Methyl 2-bromoacetate (14 g, 95 mM, 9 mL, 2 eq) was added dropwise at 25 ˚C and the mixture stirred at 50 ˚C for 12 h. TLC showed the reaction was complete. The mixture was poured into cooled H_2O (150 mL), and extracted with EtOAc (3 x 50 mL). The combined organic layer was washed with saturated aqueous citric acid (80 mL) and brine (50 mL), successively. The organic layer dried was dried over Na_2SO_4, filtered and concentrated under reduced pressure to give a solid. The crude product was washed with MTBE (20 mL) and filtered to collect methyl 2-(6-chloro-5,7-dimethyl-3-oxo-1,4-benzoxazin-4-yl)acetate (8 g, 28 mM, 59.7% yield) as an off-white solid.

TLC (20% v/v ethyl acetate in petroleum ether): R_f = 0.30 ($KMnO_4$, UV);
^{1}H NMR ($CDCl_3$, 400 MHz): δ = 6.85 (s, 1 H), 4.50 (s, 2 H), 4.47 (s, 2 H), 3.76 (s, 3 H), 2.36 (s, 3H), 2.33 (s, 3 H).

3.3.5 Ethyl 2-(6-chloro-5, 7-dimethyl-3-oxo-1, 4-benzoxazin-4-yl) acetate (1b)

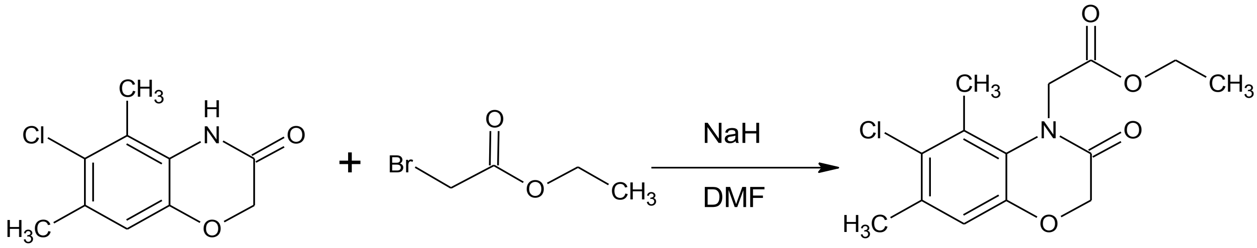

To a solution of 6-chloro-5,7-dimethyl-4H-1,4-benzoxazin-3-one (500 mg, 2 mM, 1 eq) in DMF (4 mL) was added NaH (189 mg, 5 mM, 60% purity, 2 eq) at 25 ˚C and the mixture stirred at 25 ˚C for 0.5 h. Ethyl 2-bromoacetate (789 mg, 5 mM, 523 µL, 2 eq) was added at 25 ˚C and resultant mixture stirred at 50 ˚C for 12 h. TLC showed the reaction was complete. The mixture was poured into cooled H_2O (15 mL), and the aqueous extracted with ethylacetate (2 x 30 mL). The combined organic layer was washed with saturated aqueous citric acid (30 mL) and brine (20 mL), successively, dried over Na_2SO_4, filtered and concentrated under reduced pressure to give the crude product. The residue was purified by prep-HPLC (neutral condition: column: Agela Durashell C18 150 x 25 5 µm; mobile phase: [water (10 mM NH_4HCO_3)-ACN]; B%: 42%-72%, 10.5 min). Ethyl 2-(6-chloro-5,7-dimethyl-3-oxo-1,4-benzoxazin-4-yl) acetate (108 mg, 362 µM, 15.3% yield) was obtained as an off-white solid.

TLC (20% v/v ethyl acetate in petroleum ether): $R_f = 0.30$ ($KMnO_4$, UV);
^{1}H NMR ($CDCl_3$, 400 MHz): δ = 6.85 (s, 1 H), 4.50 (s, 2 H), 4.62 (s, 2 H), 4.21 (q, 2 H, J = 7.2 Hz), 2.36 (s, 3 H), 2.33 (s, 3 H), 1.26 (t, 3 H, J = 7.2 Hz);
LCMS (method WUXIAB10): $R_t = 3.05$, $MH^+ = 298.1$.

3.3.6 Isopropyl 2-(6-chloro-5,7-dimethyl-3-oxo-1,4-benzoxazin-4-yl)acetate (1c)

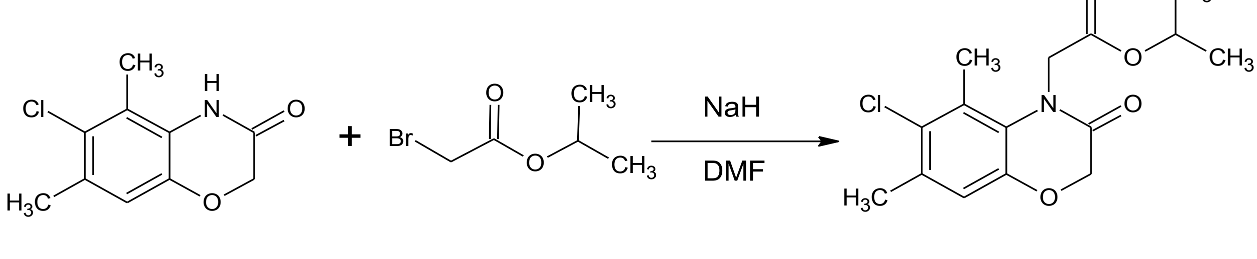

To a solution of 6-chloro-5,7-dimethyl-4H-1,4-benzoxazin-3-one (500 mg, 2 mM, 1 eq) in DMF (4 mL) was added NaH (189 mg, 5 mM, 60% purity, 2 eq) at 25 ˚C. The

mixture was stirred at 25 ˚C for 0.5 h. Then isopropyl 2-bromoacetate (855 mg, 5 mM, 611 μL, 2 eq) was added at 25 ˚C, the resulting mixture was stirred at 50 ˚C for 12 h. TLC showed the reaction was complete. The mixture was poured into cooled H_2O (15 mL), and the aqueous was extracted with ethyl acetate (30 mL × 2). The combined organic layer was washed with saturated aqueous citric acid (30 mL) and brine (20 mL), successively, the organic layer was dried over Na_2SO_4, filtered and concentrated under reduced pressure to give the crude product. The residue was purified by prep-HPLC (neutral condition: column: Agela Durashell C18 150 × 25 5 μm; mobile phase: [water (10 mM NH_4HCO_3)-ACN]; B%: 50%-70%, 10.5 min). Isopropyl 2-(6-chloro-5,7-dimethyl-3-oxo-1,4-benzoxazin-4-yl)acetate (300 mg, 963 μM, 40.7% yield) was obtained as an off-white solid.

TLC (25% v/v ethyl acetate in petroleum ether): R_f = 0.55 ($KMnO_4$, UV);
^{1}H NMR ($CDCl_3$, 400 MHz): δ = 6.84 (s, 1 H), 5.04 (sept, 1 H, J = 6.0 Hz), 4.48 (s, 2 H), 4.43 (s, 2 H), 2.47 (s, 3 H), 2.33 (s, 3 H), 1.23 (d, 6 H, J = 6.0 Hz);
LCMS (method 10-80CD): R_t = 1.28 min, MH^+ = 312.1.

3.3.7 tert Butyl 2-(6-chloro-5,7-dimethyl-3-oxo-1,4-benzoxazin-4-yl) acetate (1d)

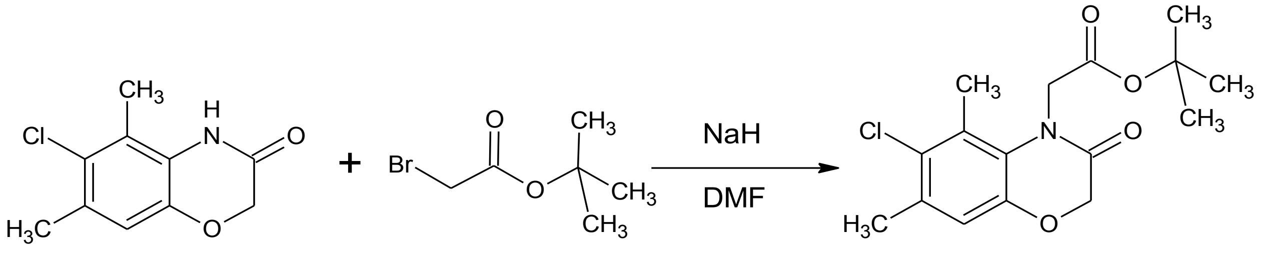

To a solution of 6-chloro-5,7-dimethyl-4H-1,4-benzoxazin-3-one (500 mg, 2 mM, 1 eq) in DMF (4 mL) was added NaH (189 mg, 5 mM, 60% purity, 2 eq) at 25 ˚C and the mixture was stirred at 25 ˚C for 0.5 h. *tert*-Butyl-2-bromoacetate (922 mg, 5 mM, 698 μL, 2 eq) was added at 25 ˚C and the resulting mixture was stirred at 50 ˚C for 12 h. TLC showed the reaction was complete. The mixture was poured into cooled H_2O (15 mL), and the aqueous was extracted with EtOAc (2 x 30 mL). The combined organic layer was washed successively with saturated aqueous citric acid (30 mL) and brine (20 mL). The organic layer was dried over Na_2SO_4, filtered and concentrated under reduced pressure to give the crude product. The residue was purified by prep-

HPLC (neutral condition: column: Agela Durashell C18 150 x 25 5 μm; mobile phase: [water(10 mM NH_4HCO_3)-ACN]; B%: 55%-75%, 10.5 min). *tert*-Butyl 2-(6-chloro-5,7-dimethyl-3-oxo-1,4-benzoxazin-4-yl) acetate (140 mg, 429 μM, 18.2% yield,) was obtained as a colorless oil.

TLC (25% v/v ethyl acetate in petroleum ether): R_f = 0.60 ($KMnO_4$, UV);

^{1}H NMR ($CDCl_3$, 400 MHz): δ = 6.84 (s, 1 H), 4.48 (s, 2 H), 4.38 (s, 2 H), 2.36 (s, 3 H), 2.33 (s, 3 H), 1.41 (s, 9 H);

LCMS (method WUXIAB10): R_t = 3.32, MH^+ ($-C_4H_9$) = 270.1.

3.3.8 2-(6-chloro-5,7-dimethyl-3-oxo-1,4-benzoxazin-4-yl)acetic acid

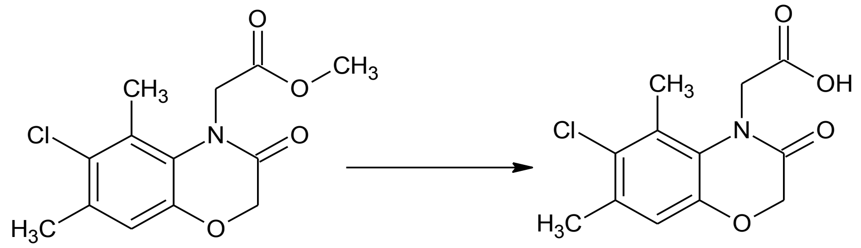

To a solution of methyl 2-(6-chloro-5,7-dimethyl-3-oxo-1,4-benzoxazin-4-yl)acetate (8 g, 26 mM, 1 eq) in THF (40 mL) was added a solution of LiOH•H_2O (1 g, 32 mM, 1.2 eq) in H_2O (10 mL) and the mixture stirred at 25 ˚C for 12 h. TLC showed the reaction was complete. The mixture was diluted with EtOAc (40 mL), the organic layer separated and the aqueous adjusted to pH~3 with HCl (6 M). The aqueous layer was further extracted with EtOAc (3 x 40 mL), dried over Na_2SO_4, filtered and concentrated under reduced pressure to give the product. 2-(6-chloro-5,7-dimethyl-3-oxo-1,4-benzoxazin-4-yl) acetic acid (7 g, 26 mM, 98.2% yield) was obtained as an off-white solid.

TLC (25% v/v ethyl acetate in petroleum ether): R_f = 0.0 ($KMnO_4$, UV);

^{1}H NMR ($CDCl_3$, 400 MHz): δ = 6.85 (1 H, s), 4.50 (2 H, s), 4.49 (2 H, s), 2.38 (3 H, s), 2.31 (3 H, s).

3.3.9 2-(6-chloro-5,7-dimethyl-3-oxo-1,4-benzoxazin-4-yl)-N,N-dimethyl-acetamide (1e)

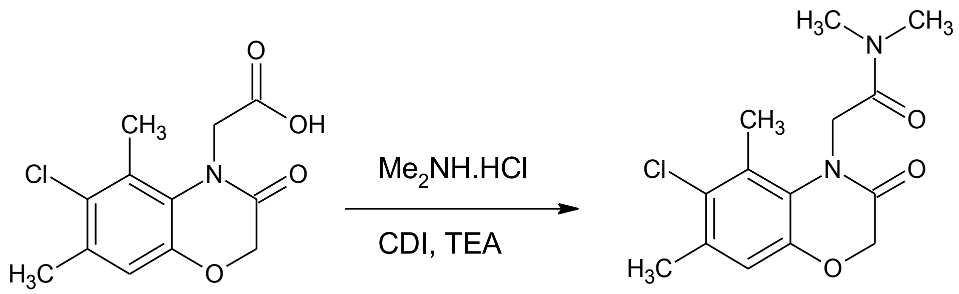

To a solution of 2-(6-chloro-5,7-dimethyl-3-oxo-1,4-benzoxazin-4-yl)acetic acid (400 mg, 1 mM, 1 eq) in DCM (5 mL) was added CDI (361 mg, 2 mM, 1.5 eq), TEA (225 mg, 2 mM, 310 µL, 1.5 eq) and dimethylamine hydrochloride (145 mg, 2 mM, 1.2 eq) and the mixture stirred at 25 ˚C for 2 h. LCMS showed the reaction was complete. The mixture was diluted with H_2O (10 mL) and extracted with EtOAc (3 x 10 mL) and concentrated under reduced pressure. The residue was purified by prep-HPLC (column: Agela Durashell C18 150 x 25 5µm; mobile phase: [water(10 mM NH_4HCO_3)-ACN]; B%: 25%-55%, 10.5 min) to give 2-(6-chloro-5,7-dimethyl-3-oxo-1,4-benzoxazin-4-yl)-*N*,*N*-dimethyl-acetamide (101 mg, 340 µM, 22.9% yield,) as a white solid.

^{1}H NMR (CD_3OD, 400 MHz): δ = 6.89 (s, 1 H), 4.63 (s, 2H), 4.49 (s, 2 H), 3.10 (s, 3 H), 2.97 (s, 3 H), 2.39 (s, 3 H), 2.32 (s, 3 H);
LCMS (method 10-80CD): R_t = 1.10 min, MH^+ = 297.1.

3.3.10 2-(6-chloro-5,7-dimethyl-3-oxo-1,4-benzoxazin-4-yl)-N-methyl-acetamide (1f)

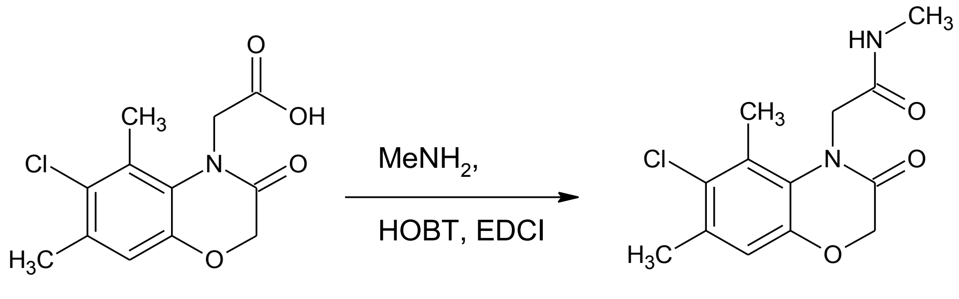

To a solution of 2-(6-chloro-5,7-dimethyl-3-oxo-1,4-benzoxazin-4-yl)acetic acid (400 mg, 1 mM, 1 eq) in DMF (5 mL) was added HOBt (301 mg, 2 mM, 1.5 eq) and EDCI (427 mg, 2 mM, 1.5 eq) at 25 ˚C. Methylamine (2 M, 1.5 mL, 2 eq) was added and the mixture was stirred at 25 ˚C for 12 hours. LCMS showed the reaction was complete. The mixture was diluted with H_2O (10 mL) and extracted with EtOAc (2 x

10 mL) and the organic layers were concentrated under reduced pressure to give a crude product. The residue was purified by prep-HPLC (column: Agela Durashell C18 150 x 25 5 µm; mobile phase: [water(10 mM NH_4HCO_3)-ACN]; B%: 25%-55%, 10.5 min) to give 2-(6-chloro-5,7-dimethyl-3-oxo-1,4-benzoxazin-4-yl)-N-methyl-acetamide (120 mg, 424 µM, 28.6% yield, 99.8% purity) as a white solid.

^{1}H NMR (CD_3OD, 400 MHz): δ = 6.90 (s, 1 H), 4.49 (s, 1 H), 4.37 (s, 1 H), 2.76 (s, 3 H), 2.40 (s, 3 H), 2.33 (s, 3 H);
LCMS (method WUXIAB10): R_t = 2.51 min. MH^+ = 283.1.

3.3.11 2-(6-chloro-5,7-dimethyl-3-oxo-1,4-benzoxazin-4-yl)acetamide (1g)

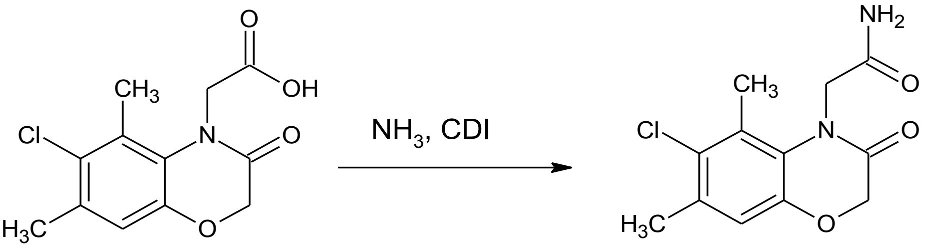

To 2-(6-chloro-5,7-dimethyl-3-oxo-1,4-benzoxazin-4-yl)acetic acid (400 mg, 1 mM, 1 eq) in DCM (5 mL) was added CDI (361 mg, 2 mM, 1.5 eq) and NH_3 (4 M in THF, 371 µL) at 25 °C. The mixture was stirred at 25 °C for 2 h. LCMS showed the reaction was complete. The mixture was diluted with H_2O (10 mL) and extracted with EtOAc (3 x 10 ml) and the organic layers concentrated under reduced pressure. The residue was purified by prep-HPLC (column: Agela Durashell C18 150 x 25 5 µm; mobile phase: [water(10 mM NH_4HCO_3)-ACN]; B%: 20%-55%, 10.5 min). 2-(6-chloro-5,7-dimethyl-3-oxo-1,4-benzoxazin-4-yl)acetamide (150 mg, 558 µM, 37.6% yield) was obtained as a white solid.

^{1}H NMR (MeOD, 400 MHz): δ = 6.89 (s, 1 H), 4.49 (s, 2 H), 4.41 (s, 2 H), 2.42 (s, 3 H), 2.32 (s, 3 H);
LCMS (method 10-80CD): R_t = 1.04 min. MH^+ = 269.1.

3.3.12 2-(6-chloro-5,7-dimethyl-3-oxo-1,4-benzoxazin-4-yl)acetonitrile

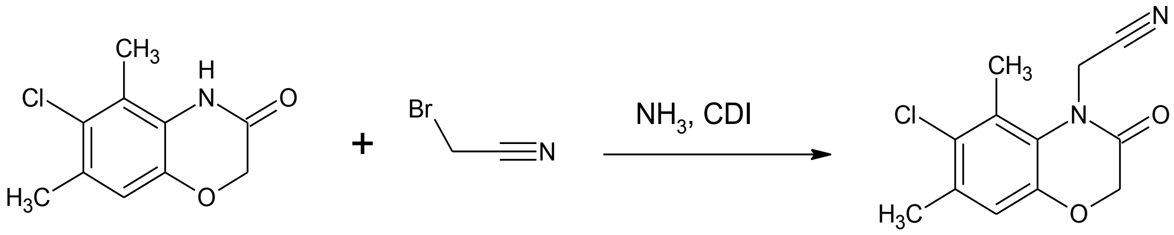

To a solution of 6-chloro-5,7-dimethyl-4H-1,4-benzoxazin-3-one (500 mg, 2 mM, 1 eq) in DMF (7 mL) was added K_2CO_3 (816 mg, 6 mM, 2.5 eq) followed by 2-bromoacetonitrile (425 mg, 4 mM, 236 μL, 1.5 eq) and the mixture stirred for at 25 ˚C 12 h. TLC and LCMS showed the reaction was complete. The resulted mixture was poured into cooled H_2O (15 mL), and the aqueous extracted with EtOAc (2 x 30 mL). The combined organic layer was washed with saturated aqueous citric acid (30 mL) and then brine (20 mL), dried over Na_2SO_4, filtered and concentrated under reduced pressure to give the crude product. The residue was purified by column chromatography (SiO_2, Petroleum ether/Ethyl acetate=20/1 to 2:1) to give 2-(6-chloro-5,7-dimethyl-3-oxo-1,4-benzoxazin-4-yl)acetonitrile (399 mg, 2 mM, 67.5% yield) as a white solid.

TLC (25% v/v ethyl acetate in petroleum ether): $R_f = 0.40$ ($KMnO_4$, UV);
^{1}H NMR ($CDCl_3$, 400 MHz): δ = 6.87 (s, 1 H), 4.59 (s, 2 H), 4.51 (s, 2 H), 2.50 (s, 3 H), 2.35 (s, 3 H);
LCMS (method 5-95AB): $R_t = 0.76$ min. $MH^+ = 251.1$.

3.3.13 6-chloro-5,7-dimethyl-4-(2H-tetrazol-5-ylmethyl)-1,4-benzoxazin-3-one (1h)

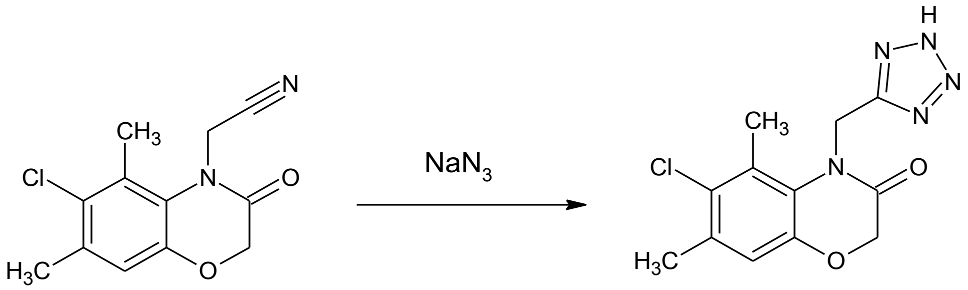

To a mixture of 2-(6-chloro-5,7-dimethyl-3-oxo-1,4-benzoxazin-4-yl)acetonitrile (650 mg, 3 mM, 1 eq) in DMF (5 mL) was added NH_4Cl (832 mg, 16 mM, 544 μL, 6 eq) followed by sodium azide (674 mg, 10 mM, 4 eq) at 25 ˚C. Then the resulting mixture was stirred at 100 ˚C for 12 h. LCMS showed the reaction was complete. The mixture

was diluted with EtOAc (40 mL), washed with H_2O (30 mL) and brine (20 mL), dried over Na_2SO_4, filtered and concentrated under reduced pressure. The residue was purified by prep-HPLC (neutral condition: column: Agela Durashell C18 150 x 25 5 µm; mobile phase: [water(10 mM NH_4HCO_3)-ACN]; B%: 10%-40%, 10.5 min) to give 6-chloro-5,7-dimethyl-4-(2H-tetrazol-5-ylmethyl)-1,4-benzoxazin-3-one (55 mg, 187 µM, 7.2% yield) as a white solid.

TLC (25% v/v ethyl acetate in petroleum ether): $R_f = 0.40$ ($KMnO_4$, UV);
^{1}H NMR (CD_3OD, 400 MHz): δ = 6.83 (s, 1 H), 5.26 (s, 2 H), 4.51 (s, 2 H), 2.37 (s, 3 H), 2.28 (s, 3 H);
LCMS (method WUXIAB10): $R_t = 2.50$ min. $MH^+ = 294.0$.

3.4 Cheminformatic analysis of biochemical entities with structures similar to that of the chosen KMO inhibitor.

The similarities between the KMO inhibitors and natural metabolites was performed[11] as follows. Structures of the candidate inhibitors, and of the list of human metabolites extracted from Recon 2[12], were encoded as a string of 1s and 0s using the MACCS166 encoding[13]. The similarity between the KMO inhibitor and each Recon2 metabolite was calculated as their Jaccard or Tanimoto similarity, i.e. the intersection over the union of bits, and rank-ordered. The most similar molecule to the new KMO inhibitor was thereby deemed to be riboflavin. Kynurenine was not ranked as highly since it is considerably smaller than riboflavin, and the Tanimoto similarity can also depend on the sizes of the comparator molecules[14,15]. However, we also recognised the potential structural similarities between the KMO inhibitors and kynurenine. The maximum common substructure of riboflavin and of **1** and its prodrug derivatives **1a**, **1b** and **1c**, is given in Fig S13B.

3.5 Steady-state enzymatic assays.

Initial screening assays and determination of IC_{50} values for each potential inhibitor compound were carried out using either 0.1 µM *Pf*KMO or 0.5 µM *Hs*KMO at fixed concentrations of L-KYN (250 µM) and NADPH (200 µM) in reaction buffer (*Hs*KMO: 20 mM potassium phosphate pH 8.0, 7 mM 2-mercaptoethanol; *Pf*KMO: 20 mM HEPES, 10 mM sodium acetate, pH 7.5, 2 mM DTT). KMO activity was measured in the presence of a range of inhibitor concentrations by monitoring the

consumption of NADPH at 340 nm upon addition of enzyme via a Cary 50 UV-Vis spectrophotometer (Agilent Technologies). Rates were determined from linear regression fits to the initial linear portion of the curves. All measurements were performed in triplicate. The IC_{50} values for inhibitors were obtained by plotting the percentage inhibition versus the inhibitor concentration and fitting to the Morrison equation[16] (equation 1) using Origin 9.0 Software (OriginLab, Northampton, MA).

$$\frac{v_i}{v_0} = 1 - \frac{\left([E] + [I] + K_i^{app}\right) - \sqrt{\left([E] + [I] + K_i^{app}\right)^2 - 4[E][I]}}{2[E]} \quad (1)$$

where v_0 is the enzyme activity without inhibitor, v_i is the enzyme activity with ligand concentration [I], [E] is the enzyme concentration, [I] is the inhibitor concentration, K_i^{app} is the apparent inhibition constant and $IC_{50} = K_i^{app} + \frac{[E]}{2}$. If $K_i^{\mathrm{app}} \gg \frac{[E]}{2}$, $IC_{50} \approx K_i^{\mathrm{app}}$.

$$K_i^{app} = K_i\left(1 + \frac{[S]}{K_m}\right) \quad (2)$$

where K_i is the inhibition constant, [S] is the substrate concentration used in the assay and K_m is the Michaelis-Menten constant[17]. The equation (2) shows the relation between K_i and K_i apparent values, as the conditions for measuring IC_{50} are different, K_i values can be used to compare with other reported KMO inhibitors.

Steady-state kinetic parameters for *Pf*KMO were calculated by measuring initial rates at varying concentrations of NADPH (5-200 μM) and L-KYN (10-250 μM). Plots of the observed rate of turnover (v) versus the varied substrate concentration were fitted to equation 2 using Origin 9.0 Software (OriginLab, Northampton, MA).

$$v = \frac{V_{\max}[A][B]}{[A][B] + K_{\mathrm{m}}^{\mathrm{A}}[B] + K_{\mathrm{m}}^{\mathrm{B}}[A] + K_i^{\mathrm{A}} K_{\mathrm{m}}^{\mathrm{B}}} \quad (3)$$

where v is the initial rate of activity, V_{max} is the maximum velocitie of the reaction, [A] and [B] are the substrate concentration, $K_{\mathrm{m}}^{\mathrm{A}}$ and $K_{\mathrm{m}}^{\mathrm{B}}$ are the Michaelis-Menten constant for different substrate and K_i^{A} is the inhibition (binding) constant of substrate A to the free enzyme while forming transitory complexes.[18]

Product inhibition studies were carried out by measuring reaction rates at varying concentrations of 3-HK and NADP at saturating and sub-saturating concentrations of one substrate and varying concentrations of the other substrate. Inhibition studies with target compounds were carried out by measuring reaction rates at varying inhibitor concentrations at saturating concentrations of one substrate and varying concentrations of the other substrate. Lineweaver-Burke plots of $1/v$ against 1/[S] were used to distinguish between different types of inhibition. The data were fitted to a straight line defined by the Lineweaver-Burke equation (equation 4), with the y intercept providing $1/V_{\max}$ and the x intercept giving $-1/K_m$.

$$\frac{1}{v} = \frac{K_m}{V_{\max}}\left(1 + \frac{[I]}{K_i}\right)\frac{1}{[S]} + \frac{1}{V_{\max}} \tag{4}$$

where v is the initial rate of activity, K_m is the Michaelis-Menten constant, V_{max} is the maximum velocity of the reaction, [I] is the inhibitor concentration, K_i is the inhibition constant and [S] is the substrate concentration.[18]

3.6 Assessment of the *in vitro* ADME parameters for KMO inhibitors

3.6.1 Analytical conditions

A Waters Micromass Quattro (serial number: QAA668) was used for analysis of the kinetic solubility samples.Waters TQD mass spectrometers (serial numbers: QBB799, QBB873 and QBB878) were used for the other sample analysis. The settings of the electrospray ion source used for data acquisition and the chromatography parameters are detailed in Tables below.

Instrument parameters for analysis

Parameter	Setting	
	Micromass Quattro	TQD
Serial number	QAA668	QBB799/873/878
Capillary voltage (kV)	3	3.5
Extractor voltage (V)	3	3
Source temp (°C)	120	150

Desolvation gas temp (°C)	350	500
Desolvation gas flow (L/h)	350	900
Cone gas flow (L/h)	100	50

Chromatographic conditions used for kinetic solubility analysis

Parameter	Setting	
Column	Zorbax SB C8 30 x 4.6 mm 3 µm	
Flow rate	1.3 mL/min	
Injection volume	10 µL	
Mobile phase	A:0.01% formic acid in water<br>B:0.01% formic acid in methanol	
Gradient profile	Time (minutes)	Mobile phase B (%)
	0.0	5
	2.5	95
	3.5	95
	3.6	5
	4.0	5

Chromatographic conditions used for hepatic microsome stability and MDCK permeability analysis

Parameter	Setting	
Column	Kinetex XB-C18 100A<br>2.1 x 50 mm 2.6 µm	
Flow rate	0.8 mL/min	
Injection volume	2 µL	
Mobile phase	A: 0.01% formic acid in water<br>B: 0.01% formic acid in ACN	
Gradient profile	Time (minutes)	Mobile phase B (%)
	0.00	5
	0.50	95
	0.85	95
	0.90	5
	1.10	5

3.6.2 Kinetic solubility

Kinetic solubility assays were performed as described in the BioFocus Standard Operating Procedure, ADMESOP-01. Using a 10 mM stock solution of each test and control compound (hydrocortisone, reserpine) in 100 % DMSO, dilutions were

prepared to a theoretical concentration of 200 μM in both 0.1 M potassium phosphate buffer containing 0.8 % NaCl (PBS), pH 7.4 (2 % DMSO final), and in 100 % DMSO. An aliquot of the 200 μM DMSO solution was then further diluted to 10 μM and all dilutions (n = 2, in 96-well plates) allowed to equilibrate at room temperature on an orbital shaker for 2 hours. The PBS dilutions were filtered using a Multiscreen HTS solubility filter plate (Millipore) and filtrate was analysed by LC-UV with confirmation of the peak of interest by mass spectrometry. The concentration of compound in PBS filtrate was determined by comparing the UV absorbance peak with that of the two DMSO dilutions as calibration standards.

The effective range of the assay is 10 - 200 μM and compounds returning values close to the upper limit may have much higher solubilities. Note that a direct correlation between thermodynamic aqueous solubility measured for a compound and kinetic 2 % DMSO solubility, as measured here, is not expected. However, it is considered that good solubility in 2 % DMSO (in the upper range of this assay) should facilitate the generation of reliable *in vitro* and *in vivo* data.

3.6.3 Compound stability in mouse hepatic microsomes and rat hepatocytes

A microsomal stability assay was performed as described in the BioFocus Standard Operating Procedure ADME-SOP-83, using pooled hepatic mouse (Xenotech/1210302) microsomes. Test and control compounds (midazolam, dextromethorphan), were prepared in DMSO and incubated at an initial concentration of 1 μM (0.25 % DMSO final, n=2) with microsomes (0.25 mg protein/mL) at 37 ˚C in the presence of the cofactor, NADPH (1 mM). Aliquots were removed at 0, 5, 10, 20 and 40 minutes for termination of reactions and compound extraction with ACN containing an analytical internal standard. Samples were centrifuged and the supernatant fractions analysed for parent compound by mass spectrometry (LC-MS/MS). The amount of compound remaining (expressed as %) was determined from the MS response in each sample relative to that in the T = 0 samples (normalised for internal standard). Ln plots of the % remaining were used to determine the half-life for compound disappearance using the relationship: $t_{1/2}$ (min) = -0.693/λ, where λ is the slope of the Ln % remaining vs time curve. The *in vitro* intrinsic clearance Cl_{int} (μL/min/mg microsomal protein), was calculated using the formula: Cl_{int} = 0.693 x 1/$t_{1/2}$ (min) x (1/mg of microsomal protein/mL) x 1000.

Rat hepatocyte compound stability assays were performed using cryopreserved vials of rat cryopreserved hepatocytes (Life Technologies), which were thawed according to manufacturer's instructions and resuspended in Williams Medium E (WME) containing cell maintenance supplement pack (CM4000, Life Technologies). Hepatocytes were incubated in suspension (0.5 million cells/mL) in 48 well non-collagen coated cell culture plates for 10 minutes at 37°C, 5% CO_2. Upon addition of an equal volume of supplemented WME containing 1 mM test compound, an aliquot of incubation solution was removed to acetonitrile containing internal standard (final concentration 0.5 mM test compound and a cell density of 0.25 million cells/mL). Similarly, aliquots were removed at 3,6,9,15,30,45,60,90 and 120 minutes. 100 mL of 80:20 water:acetonitrile was added to all samples and the analysis plate was centrifuged for 10 minutes at room temperature prior to injection and analysis of samples by UPLC-MS/MS using a Xevo TQs micro (Waters corporation, USA). XLfit (IDBS, UK) was used to calculate the exponential decay and consequently the rate constant (k) from the ratio of peak area of test compound to internal standard at each timepoint. The rate of intrinsic clearance (CLi) was then calculated. Verapamil (0.5 µM) was used as a positive control to confirm acceptable assay performance.

3.6.4 MDR1-MDCK: effective efflux ratio

The MDR1-MDCK effective efflux assay was performed as described in the BioFocus Standard Operating Procedure, ADME-SOP-56. Both wild-type and MDR1-MDCK cells (Solvo Biotechnology) were seeded onto 24-well Transwell plates at 2.35 x 105 cells per well and used in confluent monolayers after a 3 day culture at 37 ˚C under 5 % CO_2. For both cell types, test and control compounds (propranolol, vinblastine) were added (10 µM, 0.1 % DMSO final, n = 2) to donor compartments of the Transwell plate assembly in assay buffer (Hanks balanced salt solution supplemented with 25 mM HEPES, adjusted to pH 7.4) for both apical to basolateral (A>B) and basolateral to apical (B>A) measurements. Incubations were performed at 37 ˚C, with samples removed from both donor and acceptor chambers at T = 0 and 1 hour and compound analysed by mass spectrometry (LC-MS/MS) including an analytical internal standard.

Apparent permeability (P_{app}) values were determined from the relationship:
P_{app} = [$Compound_{Acceptor\ T=end}$] x $V_{Acceptor}$ / ([$Compound_{Donor\ T=0}$] x V_{Donor}) / incubation time x V_{Donor} / Area x 60 x 10^{-6} cm/s

Where V is the volume of each Transwell compartment (apical 125 μL, basolateral 600 μL), and concentrations are the relative MS responses for compound (normalized to internal standard) in the donor chamber before incubation and acceptor chamber at the end of the incubation. Area = area of cells exposed for drug transfer (0.33 cm^2). Efflux ratios (P_{app} B>A / P_{app} A>B) were calculated for each compound from the mean P_{app} values in each direction for both wild-type and MDR1-MDCK cells. The MDR1-MDCK cell line has been engineered to overexpress the efflux transporter, MDR1 (P-glycoprotein), and a finding of good permeability B>A, but poor permeability A>B, suggests that a compound is a substrate for this transporter. In order to confirm the involvement of MDR1 in any efflux seen, an "effective efflux ratio" (EER) was calculated by comparing compound efflux ratios (ER) in the two cell types, i.e. EER = ER (MDR1-MDCK)/ ER (wild-type MDCK). This ratio illustrates the effect of the over-expressed MDR1 normalised for the background movement of compound through the wild-type cells.

Lucifer Yellow (LY) was added to the apical buffer in all wells to assess viability of the cell layer. As LY cannot freely permeate lipophilic barriers, a high degree of LY transport indicates poor integrity of the cell layer and wells with a LY P_{app} > 10 x 10^{-6} cm/s were rejected. Note that an integrity failure in one well does not affect the validity of other wells on the plate.

Compound recovery from the wells was determined from MS responses (normalized to internal standard) in donor and acceptor chambers at the end of incubation compared to response in the donor chamber pre-incubation. Recoveries <50% suggest compound solubility, stability or binding issues in the assay, which may reduce the reliability of a result.

3.7 KMO activity in rat brain and liver

Two month-old male Sprague-Dawley rats were euthanized, and their brain and liver were rapidly removed and placed on ice. For the determination of KMO activity,

tissues were diluted (brain: 1:10, w/v; liver: 1:10,000, w/v) with Krebs buffer containing 100 mM Tris-HCl (pH 8.1), 10 mM KCl and 1 mM EDTA, and sonicated. 80 µl of the resulting tissue homogenates were incubated for 40 min at 37°C with 20 µl aliquots of aqueous solutions of the test compounds (or water in controls), 10 µM kynurenine, 1 mM NADPH, 3 mM glucose-6-phosphate and 1U/ml glucose-6-phosphate dehydrogenase in a total volume of 200 µl. The reaction was terminated by the addition of 50 µl of 6% perchloric acid, the denatured proteins were removed by centrifugation, and newly synthesized 3-HK was quantified by HPLC with electrochemical detection (Coulochem 5100A; ESA, Chelmsford, MA) at an oxidation potential of +0.2 mV[19].

3.8 Brain Tissue Binding

The methodology employed was a modification of that reported previously[20]. In brief, a 96 well equilibrium dialysis apparatus was used to determine the free fraction in the brain for inhibitor **1** and prodrug **1b** (HT Dialysis LLC, Gales Ferry, CT). Membranes (12-14kDA cut-off) were conditioned in deionised water for 60 minutes, followed by conditioning in 80:20 deionised water:ethanol for 20 minutes, and then rinsed in artificial cerebrospinal fluid (CSF) before use. Mouse brain was removed from the freezer and allowed to thaw on the day of experiment. Thawed brain tissue was homogenised with artificial CSF to a final composition of 1:2 brain:artificial CSF using a Covaris S2 (K Biosciences, Hoddesdon, UK). Diluted brain homogenate was spiked with the test compound (10 µg/g), and 150 µL aliquots (n = 6 replicate determinations) loaded into the 96-well equilibrium dialysis plate. Dialysis vs artificial CSF (150 µL) was carried out for 5 hours in a temperature controlled incubator at *ca.* 37ºC (Barworld scientific Ltd, UK) using an orbital microplate shaker at 125 revolutions/minute (Barworld scientific Ltd, UK). At the end of the incubation period, aliquots of brain homogenate or artificial CSF were transferred to micronic tubes (Micronic B.V., the Netherlands) and the composition in each tube balanced with control fluid, such that the volume of artificial CSF to brain is the same. Sample extraction was performed by the addition of 400 µL of acetonitrile containing an appropriate internal standard. Samples were allowed to mix

for 1 minute and then centrifuged at 3000 rpm in 96-well blocks for 15 minutes (Allegra X12-R, Beckman Coulter, USA). All samples were analysed by means of UPLC/MS/MS on a Xevo TQs micro Mass Spectrometer (Waters Corporation, USA). The unbound fraction was determined as the ratio of the peak area in artificial CSF to that in brain, with correction for dilution factor according to equation (5)[21],

$$\text{Undiluted } f_u = \frac{1/D}{((1/f_{u,apparent}) - 1) + 1/D} \qquad (5)$$

where D = dilution factor in brain homogenate and $f_{u,apparent}$ is the measured free fraction of diluted brain homogenate.

3.9 Plasma protein binding

In brief, a 96 well equilibrium dialysis apparatus was used to determine the free fraction in plasma for inhibitor **1** (HT Dialysis LLC, Gales Ferry, CT). Membranes (12-14 kDA cut-off) were conditioned in deionised water for 60 minutes, followed by conditioning in 80:20 deionised water:ethanol for 20 minutes, and then rinsed in isotonic buffer before use. Female CD1 mouse plasma was removed from the freezer and allowed to thaw on the day of experiment. Thawed plasma was then centrifuged (Allegra X12-R, Beckman Coulter, USA), spiked with test compound (final concentration 10 ug/mL), and 150 µL aliquots (n = 6 replicate determinations) loaded into the 96-well equilibrium dialysis plate. Dialysis vs isotonic buffer (150 µL) was carried out for 5 hours in a temperature controlled incubator at *ca.* 37ºC (Barworld scientific Ltd, UK) using an orbital microplate shaker at 100 revolutions/minute (Barworld scientific Ltd, UK). At the end of the incubation period, 50 uL aliquots of plasma or buffer were transferred to micronic tubes (Micronic B.V., the Netherlands) and the composition in each tube balanced with control fluid (50 µL), such that the volume of buffer to plasma is the same. Sample extraction was performed by the addition of 200 µL of acetonitrile containing an appropriate internal standard. Samples were allowed to mix for 1 minute and then centrifuged at 3000 rpm in 96-well blocks for 15 minutes (Allegra X12-R, Beckman Coulter, USA) after which 150 µL of supernatant was removed to 50 µL of water.. All samples were analysed by UPLC-MS/MS on a Xevo TQs micro Mass Spectrometer (Waters Corporation, USA). The unbound fraction was determined as the ratio of the peak area in buffer to that in plasma.

1.10 Rat hepatocyte stability

A vial of rat cryopreserved hepatocytes, supplied by Life Technologies, was thawed according to manufacturer's instructions and cells resuspended in Williams Medium E (WME) containing cell maintenance supplement pack (CM4000, Life Technologies). Hepatocytes were incubated in suspension (0.5 million cells/mL) in 48 well non-collagen coated cell culture plates for 10 minutes at 37°C, 5% CO_2. Upon addition of an equal volume of supplemented WME containing 1 mM test compound, an aliquot of incubation solution was removed to acetonitrile containing internal standard (final concentration 0.5 mM test compound and a cell density of 0.25 million cells/mL). Similarly, aliquots were removed at 3,6,9,15,30,45,60,90 and 120 minutes. 100 mL of 80:20 water:acetonitrile was added to all samples and the analysis plate was centrifuged for 10 minutes at room temperature prior to injection and analysis of samples by UPLC-MS/MS using a Xevo TQs micro (Waters corporation, USA). XLfit (IDBS, UK) was used to calculate the exponential decay and consequently the rate constant (k) from the ratio of peak area of test compound to internal standard at each timepoint. The calculated hepatocyte intrinsic clearance (CLi; mL/min/10^6 cells) was scaled to in vivo CLi (ml/min/g Liver) using the hepatocellularity scaling factor of 120 x 10^6 cells/g of liver. Verapamil (0.5 µM) was used as a positive control to confirm acceptable assay performance.

References

1 Cleland, W. W. The kinetics of enzyme-catalyzed reactions with two or more substrates or products. I. Nomenclature and rate equations. *Biochim Biophys Acta* **67**, 104-137 (1963).

2 Amaral, M. *et al.* Structural basis of kynurenine 3-monooxygenase inhibition. *Nature* **496**, 382-385 (2013).

3 *InterBioScreen Synthetic compounds library*, <http://www.ibscreen.com/index.htm>.

4 *ChemBridge database*, <http://www.chembridge.com/index.php>.

5 *Maybridge screening libraries*, <http://www.maybridge.com/>.

6 *Screening Compounds: MyriaScreen Diversity Collection*, <http://www.sigmaaldrich.com/chemistry/chemistry-services/high-throughput-screening/screening-compounds.html>.

7 Small-Molecule Drug Discovery Suite 2014-3: Phase, version 4.0, Schrödinger, LLC, New York, NY, 2014.

8 Pellicciari, R. *et al.* Modulation of the kynurine pathway of tryptophan metabolism in search for neuroprotective agents. Focus on kynurenine-3-hydroxylase. *Adv Exp Med Biol* **527**, 621-628 (2003).

9 Toledo-Sherman, L. M. *et al.* Development of a series of aryl pyrimidine kynurenine monooxygenase inhibitors as potential therapeutic agents for the treatment of Huntington's disease. *J Med Chem* **58**, 1159-1183 (2015).

10 Greenough, M. A., Camakaris, J. & Bush, A. I. Metal dyshomeostasis and oxidative stress in Alzheimer's disease. *Neurochem. Int.* **62**, 540-555 (2013).

11 S, O. H., Swainston, N., Handl, J. & Kell, D. B. A 'rule of 0.5' for the metabolite-likeness of approved pharmaceutical drugs. *Metabolomics* **11**, 323-339 (2015).

12 Thiele, I. *et al.* A community-driven global reconstruction of human metabolism. *Nat. Biotechnol.* **31**, 419-425 (2013).

13 Durant, J. L., Leland, B. A., Henry, D. R. & Nourse, J. G. Reoptimization of MDL keys for use in drug discovery. *J Chem Inf Comp Sci* **42**, 1273-1280 (2002).

14 Flower, D. R. On the properties of bit string-based measures of chemical similarity. *J Chem Inf Comp Sci* **38**, 379-386 (1998).

15 O'Hagan, S. & Kell, D. B. MetMaxStruct: A Tversky-Similarity-Based Strategy for Analysing the (Sub)Structural Similarities of Drugs and Endogenous Metabolites. *Front Pharmacol* **7**, 266 (2016).

16 Morrison, J. F. Kinetics of the reversible inhibition of enzyme-catalysed reactions by tight-binding inhibitors. *Biochim Biophys Acta* **185**, 269-286 (1969).

17 Breinbauer, R. Evaluation of Enzyme Inhibitors in Drug Discovery. Von Robert A. Copeland. *Angew Chem* **117**, 6603-6603 (2005).

18 Bisswanger, H. *Enzyme Kinetics: Principles and Methods*. (John Wiley & Sons, 2008).

19 Amori, L., Guidetti, P., Pellicciari, R., Kajii, Y. & Schwarcz, R. On the relationship between the two branches of the kynurenine pathway in the rat brain in vivo. *J Neurochem* **109**, 316-325 (2009).

20 Summerfield, S. G. *et al.* Central nervous system drug disposition: the relationship between in situ brain permeability and brain free fraction. *J Pharmacol Exp Ther* **322**, 205-213 (2007).

21 Kalvass, J. C. & Maurer, T. S. Influence of nonspecific brain and plasma binding on CNS exposure: implications for rational drug discovery. *Biopharm. Drug Dispos.* **23**, 327-338 (2002).

22 Hutchinson, J. P. *et al.* Structural and mechanistic basis of differentiated inhibitors of the acute pancreatitis target kynurenine-3-monooxygenase. *Nat. Commun.* **8**, 15827 (2017).

23 Breton, J. *et al.* Functional characterization and mechanism of action of recombinant human kynurenine 3-hydroxylase. *Eur J Biochem* **267**, 1092-1099 (2000).

24 Crozier-Reabe, K. R., Phillips, R. S. & Moran, G. R. Kynurenine 3-monooxygenase from Pseudomonas fluorescens: substrate-like inhibitors both stimulate flavin reduction and stabilize the flavin-peroxo intermediate yet result in the production of hydrogen peroxide. *Biochemistry* **47**, 12420-12433 (2008).
