# Supplementary material for: A brain-permeable inhibitor of the neurodegenerative disease target kynurenine 3-monooxygenase prevents accumulation of neurotoxic metabolites
Source: Commun Biol. 2019 Jul 24;2:271. doi: 10.1038/s42003-019-0520-5 (PMC6656724; doi:10.1038/s42003-019-0520-5)
Supplement: Supplementary file 2 — Description of Additional Supplementary Files [file 42003_2019_520_MOESM2_ESM.docx]

**Description of Additional Supplementary Files**

**File Name**: Supplementary Data

**Description**:  Source data for Figure 3b-f
