# Supplementary material for: A brain-permeable inhibitor of the neurodegenerative disease target kynurenine 3-monooxygenase prevents accumulation of neurotoxic metabolites
Source: Commun Biol. 2019 Jul 24;2:271. doi: 10.1038/s42003-019-0520-5 (PMC6656724; doi:10.1038/s42003-019-0520-5)
Supplement: Supplementary file 3 — Reporting Summary [file 42003_2019_520_MOESM3_ESM.pdf]

natureresearch

Corresponding author(s): Nigel Scrutton

Last updated by author(s): Jun 26, 2019

Reporting Summary

Nature Research wishes to improve the reproducibility of the work that we publish. This form provides structure for consistency and transparency in reporting. For further information on Nature Research policies, see Authors & Referees and the Editorial Policy Checklist.

Statistics

For all statistical analyses, confirm that the following items are present in the figure legend, table legend, main text, or Methods section.

n/a	Confirmed	
☐	☒	The exact sample size (n) for each experimental group/condition, given as a discrete number and unit of measurement
☐	☒	A statement on whether measurements were taken from distinct samples or whether the same sample was measured repeatedly
☐	☒	The statistical test(s) used AND whether they are one- or two-sided<br>*Only common tests should be described solely by name; describe more complex techniques in the Methods section.*
☒	☐	A description of all covariates tested
☒	☐	A description of any assumptions or corrections, such as tests of normality and adjustment for multiple comparisons
☐	☒	A full description of the statistical parameters including central tendency (e.g. means) or other basic estimates (e.g. regression coefficient) AND variation (e.g. standard deviation) or associated estimates of uncertainty (e.g. confidence intervals)
☐	☒	For null hypothesis testing, the test statistic (e.g. F, t, r) with confidence intervals, effect sizes, degrees of freedom and P value noted<br>*Give P values as exact values whenever suitable.*
☒	☐	For Bayesian analysis, information on the choice of priors and Markov chain Monte Carlo settings
☒	☐	For hierarchical and complex designs, identification of the appropriate level for tests and full reporting of outcomes
☒	☐	Estimates of effect sizes (e.g. Cohen's d, Pearson's r), indicating how they were calculated

Our web collection on statistics for biologists contains articles on many of the points above.

Software and code

Policy information about availability of computer code

Data collection	Generic Data Acquisition (GDA), MassLynx2.1
Data analysis	Origin 9.0, Xia2, Phenix 1.13, COOT 0.8.9.1

For manuscripts utilizing custom algorithms or software that are central to the research but not yet described in published literature, software must be made available to editors/reviewers. We strongly encourage code deposition in a community repository (e.g. GitHub). See the Nature Research guidelines for submitting code & software for further information.

Data

Policy information about availability of data

All manuscripts must include a data availability statement. This statement should provide the following information, where applicable:

- Accession codes, unique identifiers, or web links for publicly available datasets
- A list of figures that have associated raw data
- A description of any restrictions on data availability

The atomic coordinates and experimental data (codes 6FP0, 6FP1, 6FPH, 6FOX, 6FOY & 6FOZ) have been deposited in the Protein Data Bank (www.wwpdb.org). Raw data used for Figure 3 are available in Supplementary Data 1. All other data are available from the corresponding author on reasonable request.

Field-specific reporting

Please select the one below that is the best fit for your research. If you are not sure, read the appropriate sections before making your selection.

☒ Life sciences ☐ Behavioural & social sciences ☐ Ecological, evolutionary & environmental sciences

Life sciences study design

All studies must disclose on these points even when the disclosure is negative.

Sample size	Sample size number was determined empirically from past experience based on previous results obtained with these particular methods approaches.
Data exclusions	No data were excluded from the analyses
Replication	At least three replicates were taken for the measurements, though in the case of two pharmacokinetic analyses only 2 of the 3 values were above the level of detection (as noted in the tables and figure legends).
Randomization	Organisms were allocated into experimental groups at random.
Blinding	Blinding was not relevant to our study. Our study is not about the efficacy of the compounds so there is no need to blind.

Reporting for specific materials, systems and methods

We require information from authors about some types of materials, experimental systems and methods used in many studies. Here, indicate whether each material, system or method listed is relevant to your study. If you are not sure if a list item applies to your research, read the appropriate section before selecting a response.

Materials & experimental systems

n/a	Involved in the study	
☒	☐	Antibodies
☐	☒	Eukaryotic cell lines
☒	☐	Palaeontology
☐	☒	Animals and other organisms
☒	☐	Human research participants
☒	☐	Clinical data

Methods

n/a	Involved in the study	
☒	☐	ChIP-seq
☐	☒	Flow cytometry
☒	☐	MRI-based neuroimaging

Eukaryotic cell lines

Policy information about cell lines

Cell line source(s)	Human K562 Cell line stored in the lab
Authentication	Cell lines used were not authenticated
Mycoplasma contamination	All cell lines tested negative for mycoplasma contamination
Commonly misidentified lines (See ICLAC register)	No commonly misidentified lines were used in the study

Animals and other organisms

Policy information about studies involving animals; ARRIVE guidelines recommended for reporting animal research

Laboratory animals	Female C57BL6 mice (6 - 12 weeks old), flies elavGAL4(c155), adult male Sprague-Dawley rats (10 weeks old), Han Wistar rats (7 - 9 weeks old)
Wild animals	The study did not involve wild animals
Field-collected samples	The study did not involve samples collected from field
Ethics oversight	The pharmacokinetics studies were performed at the Drug Discovery Unit, School of Life Sciences, University of Dundee, UK. All regulated procedures on living animals in the DDU were carried out under the authority of a project licence issued by the Home Office under the Animals (Scientific Procedures) Act 1986, as amended in 2012 (and in compliance with EU Directive EU/2010/63). Licence applications were approved by the University's Ethical Review Committee (ERC) before submission to the Home Office. The ERC has a general remit to develop and oversee policy on all aspects of the use of animals on University premises and is a sub-committee of the University Court, its highest governing body.<br>Analysis of de novo 3-HK synthesis was performed at the Maryland Psychiatric Research Center, University of Maryland, USA. Animals were housed in a temperature-controlled, AAALAC-approved animal facility on a 12/12h-light/dark cycle with unlimited

access to food and water. The experimental protocol was approved by the Institutional Animal Care and Use Committee of the University of Maryland School of Medicine.

Note that full information on the approval of the study protocol must also be provided in the manuscript.

Flow Cytometry

Plots

Confirm that:

- [ ] The axis labels state the marker and fluorochrome used (e.g. CD4-FITC).
- [x] The axis scales are clearly visible. Include numbers along axes only for bottom left plot of group (a 'group' is an analysis of identical markers).
- [ ] All plots are contour plots with outliers or pseudocolor plots.
- [ ] A numerical value for number of cells or percentage (with statistics) is provided.

Methodology

Sample preparation	K562 cells from human blood were suspended in Dulbecco's phosphate-buffered saline (DPBS) and no tissue processing steps were used, fluorescence of Riboflavin were detected directly.
Instrument	SONY SH800 flow cytometer
Software	Flowjo v10
Cell population abundance	Cell density 0.5 x 10^6 cells/mL
Gating strategy	Populations were gated on FSC-A vs SSC-A according to the gate displayed in supplementary figure

- [x] Tick this box to confirm that a figure exemplifying the gating strategy is provided in the Supplementary Information.
